# A Review of Preventative Methods against Human Leishmaniasis Infection

**DOI:** 10.1371/journal.pntd.0002278

**Published:** 2013-06-20

**Authors:** Lisa Stockdale, Robert Newton

**Affiliations:** 1 Jenner Institute, University of Oxford, Oxford, United Kingdom; 2 Department of Health Sciences, University of York, York, United Kingdom; 3 Medical Research Council/Uganda Virus Research Institute Research Unit on AIDS, Entebbe, Uganda; Institute of Tropical Medicine, Belgium

## Abstract

**Background:**

Leishmaniasis is an intracellular parasitic infection transmitted to humans via the sandfly. Approximately 350 million people are at risk of contracting the disease and an estimated 1.6 million new cases occur annually. Of the two main forms, visceral and cutaneous, the visceral form is fatal in 85–90% of untreated cases.

**Aims:**

This literature review aims to identify and evaluate the current evidence base for the use of various preventative methods against human leishmaniasis.

**Methods:**

A literature search was performed of the relevant database repositories for primary research conforming to *a priori* inclusion and exclusion criteria.

**Results:**

A total of 84 controlled studies investigating 12 outcome measures were identified, implementing four broad categories of preventative interventions: animal reservoir control, vector population control, human reservoir control and a category for multiple concurrently implemented interventions. The primary studies investigated a heterogeneous mix of outcome measures using a range of different methods.

**Conclusions:**

This review highlights an absence of research measuring human-specific outcomes (35% of the total) across all intervention categories. The apparent inability of study findings to be generalizable across different geographic locations, points towards gaps in knowledge regarding the biology of transmission of *Leishmania* in different settings. More research is needed which investigates human infection as the primary outcome measure as opposed to intermediate surrogate markers, with a focus on developing a human vaccine.

## Author Summary

Leishmaniasis is a vector-borne parasite infection, transmitted to humans by sandflies. It is estimated to cause 1.6 million new cases of disease annually. Of the two main forms, so-called “visceral” and “cutaneous”, the visceral form is fatal in 85–90% of untreated cases. This literature review provides a comprehensive summary of all the available evidence relating to the impact of interventions against infection on the burden of leishmaniasis in people and highlights the absence of high quality evidence demonstrating an effect. Four broad categories of preventative interventions are identified, investigating a range of strategies, from protection of humans against infection, to interventions aimed one stage upstream of human infection (targeting the sandfly vector), and even further, to interventions targeting animal reservoir species. Based on the current lack of understanding of the dynamics of transmission of *Leishmania*, we conclude that scant resources might be best directed toward prevention of human infections, with a focus on development of a human vaccine.

## Introduction

### *Leishmania*: Parasite and Vector

*Leishmania* is of the protozoan genus trypanosomatida. The parasite resides intracellularly causing the disease leishmaniasis, and is transmitted between hosts by the bite of the female sandfly (genus *Phlebotomus* in the Old World localities of Europe, Africa and Asia, and *Lutzomyia* in the New World – Americas and Oceania). The primary hosts are vertebrates and commonly infected animals include humans, domestic dogs and cats, opossums, the crab-eating fox and the common black rat [1].

The sandfly comprises five genera and over 700 species. Approximately 30 species are thought to be implicated in transmission of *Leishmania* parasites [2]. Sandflies are found around human settlements and breed in organic matter such as leaf litter, manure and in rodent burrows [3], and are approximately a third of the size of mosquitos, measuring between 2–3 mm in length [4]. They are often categorised by virtue of where they bite (being classified as either endophagic (biting indoors) or exophagic (biting outdoors)), as well as where they rest (either endophilic (resting indoors) or exophilic (resting outdoors)) [5].

In humans, the clinical forms of the leishmaniases are broadly categorised into visceral leishmaniasis (VL) and cutaneous leishmaniasis (CL) (cutaneous forms presenting in a spectrum ranging from cutaneous, mucosal and diffuse cutaneous) [6].

*Leishmania* occurs in five continents and is endemic in 98 countries [7]. The World Health Organisation (WHO) estimate that 350 million people are at risk of contracting leishmaniasis [6]. Approximately 58,000 cases of visceral leishmaniasis and 220,000 cutaneous cases are officially reported each year. However it is thought that only around two thirds of countries actually report incidence data, with the sparsest data from Africa [7]. Based on assessments of under-reporting, 0.2–0.4 million new cases of VL and 0.7–1.2 million new cases of CL are estimated to occur every year [7].

There are several reasons for under-reporting of official *Leishmania* cases [2];

- Most official data are compiled through passive case detection
- Passive case detection relies on individuals presenting themselves for medical attention; however since leishmaniasis is associated with poverty, many patients do not have access to health care and do not, therefore, come to medical attention
- Many cases are misdiagnosed, undiagnosed or unreported, especially when availability of diagnostic tests is poor
- Leishmaniasis is often not a legally notifiable disease
- The number of asymptomatic infections (or individuals with sub-clinical infection - which may act as a reservoir) are not reported and could be an important driver for future infections

After malaria and African trypanosomiasis (sleeping sickness), the leishmaniases are the third most important vector-borne disease and are ranked ninth in terms of global burden of disease of all infectious and parasitic diseases [8]; accounting for more than 57,000 deaths per year and an estimated 2–2.4 million Disability Adjusted Life Years (DALYs) lost [8], [9].

When discussing the eco-epidemiology of leishmaniasis, there are considered to be four main forms of disease which are named with respect to the mode of transmission; Zoonotic Visceral Leishmaniasis (ZVL), Anthroponotic Visceral Leishmaniasis (AVL), Zoonotic Cutaneous Leishmaniasis (ZCL) and Anthroponotic Cutaneous Leishmaniasis (ACL). In anthroponotic forms, which are mostly located in Old World foci, humans are considered to be the main reservoir for infection whereas in zoonotic forms (mainly in New World areas such as Brazil), animals are thought to be the major source of parasites [10].

Following the 2007 World Health Assembly, where a resolution by member states to improve research on prevention, control and management of leishmaniasis was approved, the WHO convened the Expert Committee on Leishmaniasis in March 2010. The resulting technical report “Control of the Leishmaniases” [6] was the first updated report in more than 20 years. It details recommendations for diagnosis, treatment and prevention.

Although this report makes a start on describing the problem, as well as bringing together and directing global efforts to reduce the burden of the Leishmaniases, it highlights the fact that the disease is very complicated. There are various different diagnostic modalities, treatments and preventative methods, being more or less useful depending on the *Leishmania* species, the vector characteristics, the host immunity levels, the main reservoir, as well as the socio-economic and political makeup of the locality.

The fact that the true burden of disease is not accurately known (due in part, to reliance on passive case detection, as well as an array of issues with different diagnostic modalities), further complicates any efforts to deploy efficient methods of prevention and disease management and to secure funding for research.

Human VL, is mainly caused by two species of *Leishmania* parasites, each having a characteristic regional distribution, as described by Gill & Beeching [11]

- *L. infantum* is the causative agent in the Mediterranean, Middle East, Central Asia, China and Central and South America
- *L. donovani* in India and East Africa

VL may also be caused by *L. tropica* in the Old World and *L. amazonesis* in the New World, and is fatal in 85–90% of untreated cases and up to 50% of treated cases [11].

Approximately 90% of CL occurs in Afghanistan, Pakistan, Syria, Saudi Arabia, Algeria, Islamic Republic of Iran, Brazil, and Peru [12].

CL occurs in a spectrum of clinical presentations ranging from ulceration of the skin only (cutaneous), to various degrees of mucosal involvement (diffuse cutaneous and mucocutaneous).

Distinct sub-genii of *Leishmania* are thought to cause different cutaneous clinical presentations, usually divided into Old and New World regions. However *Leishmania* species which are implicated in VL can cause cutaneous disease (and vice versa, especially when the individual has co-infections) [11].

Mortality associated with CL is not significant; however the morbidity, in the form of disfigurement, with subsequent social stigmatisation which arises from cutaneous lesions and the resulting scars is very important. In endemic areas many people have the belief that CL can be transferred through physical contact [12] resulting in restriction of social participation. Arguably, equally as important in terms of burden of disease as the health and economic effects, are the detrimental impacts on quality of life and mental health resulting from social stigma [13].

### Diagnosis

Clinical diagnosis of VL is often confused with other diseases such as malaria, schistosomiasis, African trypanosomiasis, miliary tuberculosis and malnutrition, and for CL, with tropical ulcers, leprosy and skin cancer [14].

Importantly, infection does not always result in clinical presentation of symptoms. The ratio of asymptomatic infections to clinical infections is thought to vary between 1∶2.6 to 50∶1 [15]. This presents a major problem for organisations relying on passive case detection when determining the true burden of disease and the size of the reservoir for future infections in areas of anthroponotic transmission. It is also interesting for future disease control developments to understand the genetically determined immunological factors that regulate clinical manifestation in humans [15].

The gold standard for confirmation of *Leishmania* infection is visualisation of parasites by microscopy; in a tissue smear such as a splenic aspirate, bone marrow or liver biopsy for VL [14], and scrapings or fluid from cutaneous sores in the case of CL [3]. Potential complications with obtaining tissue smears and biopsies, as well as the need for specialist medical staff and equipment mean that less invasive but equally as sensitive and specific diagnostic tools are needed for diagnosis of VL. Polymerase Chain Reaction (PCR) detection of parasite DNA in blood or organs is highly sensitive and specific but the high cost and need for specialised equipment and staff limit its use to hospitals and research centres [15].

Serological diagnostic methods are increasingly being used for diagnosis of VL but are not suitable for diagnosis of CL. One major problem with these methods is that serum antibodies remain in the body after successful treatment for several years, and therefore relapses cannot be detected using the same method [15]. Another problem is that a proportion of the population of an endemic area will test positive for serum antibodies even though they have no history of clinical leishmaniasis due to asymptomatic infection [16]. This makes it difficult to differentiate asymptomatic infected individuals from successfully treated, and from apparently cured individuals who may relapse in the future. There are a range of serologic assays including ELISA, IFAT, DAT, rK39.

More recently new techniques including loop-mediated isothermal amplification (LAMP), Nucleic Acid Sequence-Based Assay (NASBA) and Latex Agglutination Test (KAtex) have been developed but as yet have not been used in the trials reviewed here.

Skin hypersensitivity tests such as the Montnegro or Leishmanin skin test (MST/LST) are used to detect cell-mediated immunity using intradermal injection of *Leishmania* antigen. A negative response is normally seen during active infection with VL, with a switch to a positive skin test after cure. A positive result is also seen after asymptomatic infection [6]. A lack of sensitivity (14%) has been seen in LST for diagnosis of VL in India, limiting its utility [17].

The number of diagnostic tests available, as well as the variation in antibodies and antigens used in tests, coupled with the appearance of counterfeit immunochromatographic tests on the Indian subcontinent [6], shows the need for not only more reliable field-appropriate diagnostics, but standardisation and regulation of those already in use.

In areas of anthroponotic leishmaniasis, effective treatment of VL will help to decrease the human reservoir, however in areas of zoonotic transmission, treatment of humans will not solve the problem of potential human reinfection from an infected animal reservoir.

**Figure 1:**
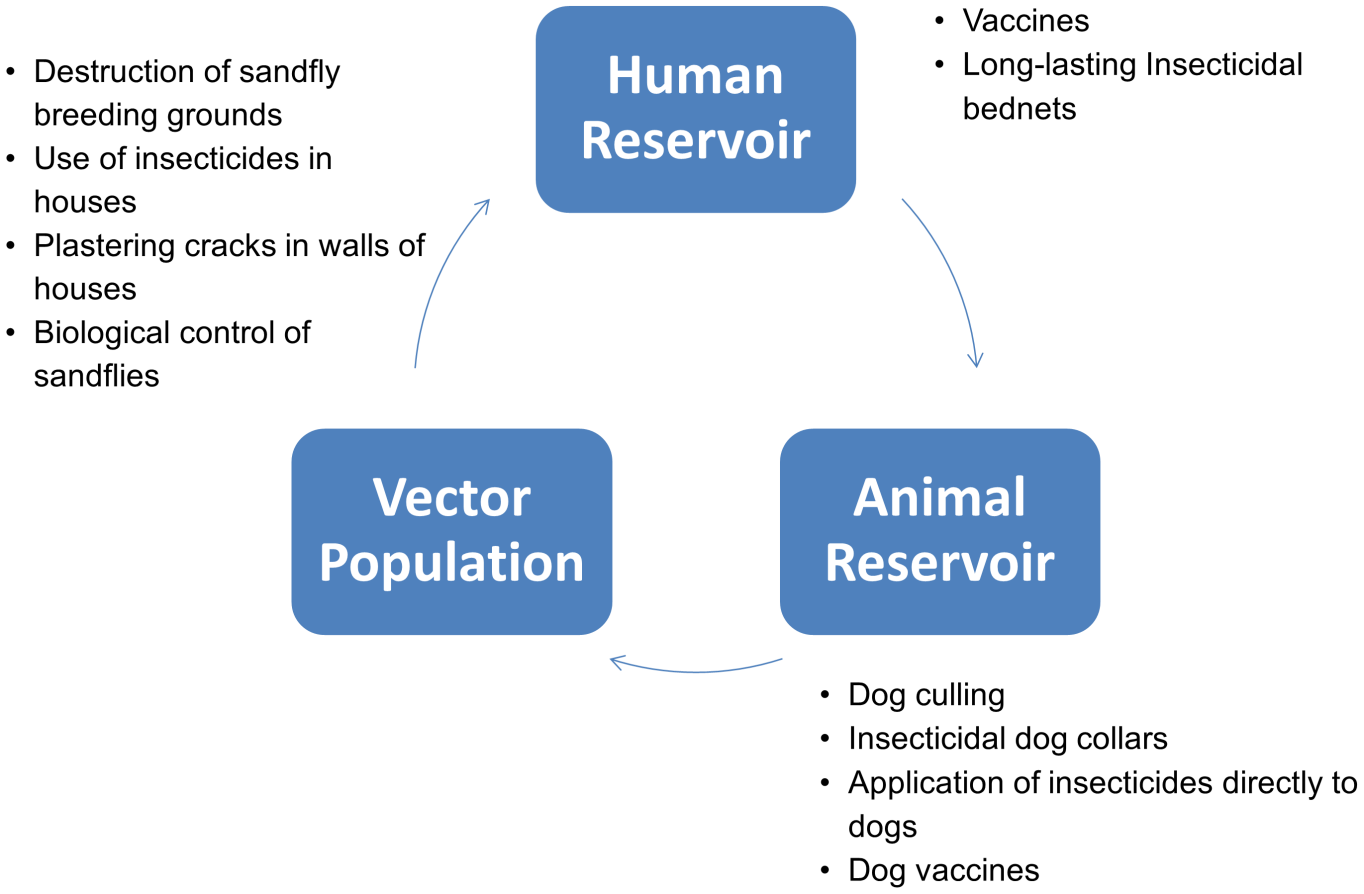
Range of preventative interventions. Potential areas of intervention include; targeting the vector population, the animal reservoir population, or attempting to either prevent humans being bitten, being infected, or from developing clinical symptoms.

With the lack of effective drugs, prohibitive treatment costs and the possibility of relapse and resistance, clearly there is a need for a more effective way to stop the cycle of infection in both zoonotic and anthroponotic transmission. Potential areas of intervention include targeting the vector or animal reservoir population, or attempting to either prevent humans being bitten, being infected, or from developing clinical symptoms. Figure 1 illustrates the potential areas of intervention.

This literature review aims to provide a comprehensive account of the methods used to prevent human infection with *Leishmania* by performing a systematic search of all interventions aimed at reducing human disease incidence.

## Methods

### Search Strategy

**Figure 2:**
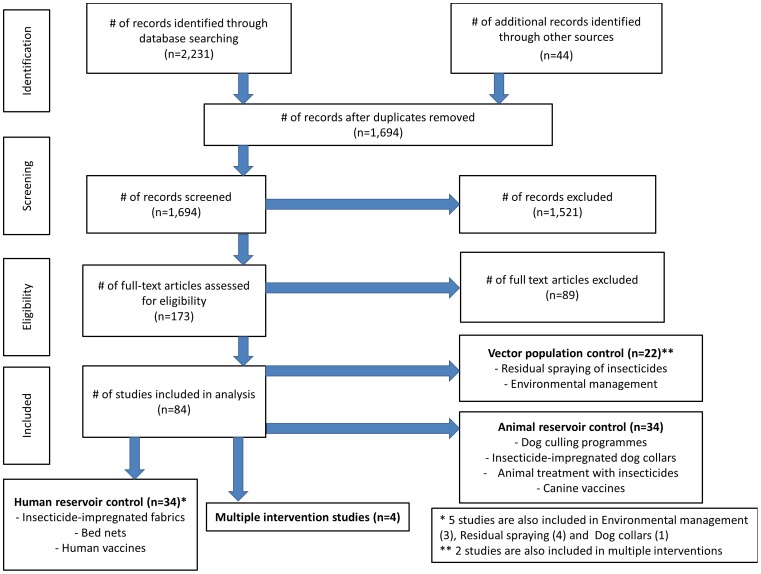
PRISMA flow chart of included studies. PRISMA flow chart documenting numbers of studies retrieved, included and excluded at each stage of the literature search.

Medline, EMBASE, CENTRAL, Web of Science, LILACS and WHOLIS were searched using terms relating to the keywords “leishmania”, “leishmaniasis”, “kala azar” (see Supplemental Materials Text S1 for full search terms for each database). Hand-searching of references of relevant studies and review articles was also performed and relevant articles retrieved. Numbers of studies retrieved, included and excluded were documented and recorded using a flow chart of stages of inclusion following the recommendations of the Preferred Reporting Items for Systematic Reviews and Meta-Analyses (PRISMA) group [18] (Figure 2).

### Inclusion and Exclusion Criteria

#### Inclusion

Only randomised controlled trials (RCTs) and controlled trials were included. Any form of leishmaniasis, and any preventative method relating to vector control, human or animal reservoir control were included. The search was not limited by date of publication and includes everything identified up until October 2012.

#### Exclusion

Treatment studies on patients with leishmaniasis were not included. Laboratory studies and vaccine studies without evidence of natural or artificial challenge with *Leishmania* were also excluded from the review. Case series, case reports, case control studies, cohort studies and economic evaluations were not included. Foreign language papers were limited to French, Spanish, Italian and Portuguese.

A total of 89 studies were excluded after ordering the full paper. The reasons for exclusion included: uncontrolled trials, human and dog vaccine trials which did not study efficacy with infection as an outcome, treatment interventions on already-infected individuals and laboratory-based studies.

### Study Selection

After all titles and abstracts resulting from the electronic and hand searching were assessed against the predetermined inclusion and exclusion criteria by use of a standardised study eligibility form, the full article was retrieved.

The full paper was assessed against the inclusion/exclusion criteria. Any paper excluded at this stage was documented with a reason for the exclusion.

In order to extract relevant data as systematically as possible, an electronic data extraction template form was used. Due to the anticipated heterogeneity of the study types and outcomes investigated, the template section headings were designed to be broad in order to capture as much information as possible.

### Data Synthesis

Based on scoping searches, it was not anticipated that meta-analysis would be possible due to the array of interventions and outcome measurements. Studies were analysed by narrative synthesis, making use of subgroup divisions based on which species (human, animal reservoir or vector) was the focus of the intervention. Within these groups there were further subdivisions based on the outcome measurements investigated. The primary measurement of interest was human infection rates. In many cases however, intermediate surrogate measurements were investigated which have differing applicability to potential changes in human incidence/prevalence of disease. Studies were grouped and analysed based on these outcome measures.

## Results and Discussion

A total of 84 studies conducted in 22 countries, across four continents, investigating 12 outcome measures, were included in this review. These studies were broadly classified into four groups of intervention types, relating to animal reservoir control, vector population control, human reservoir control and finally a group that includes studies where multiple interventions were conducted concurrently.

**Figure 3:**
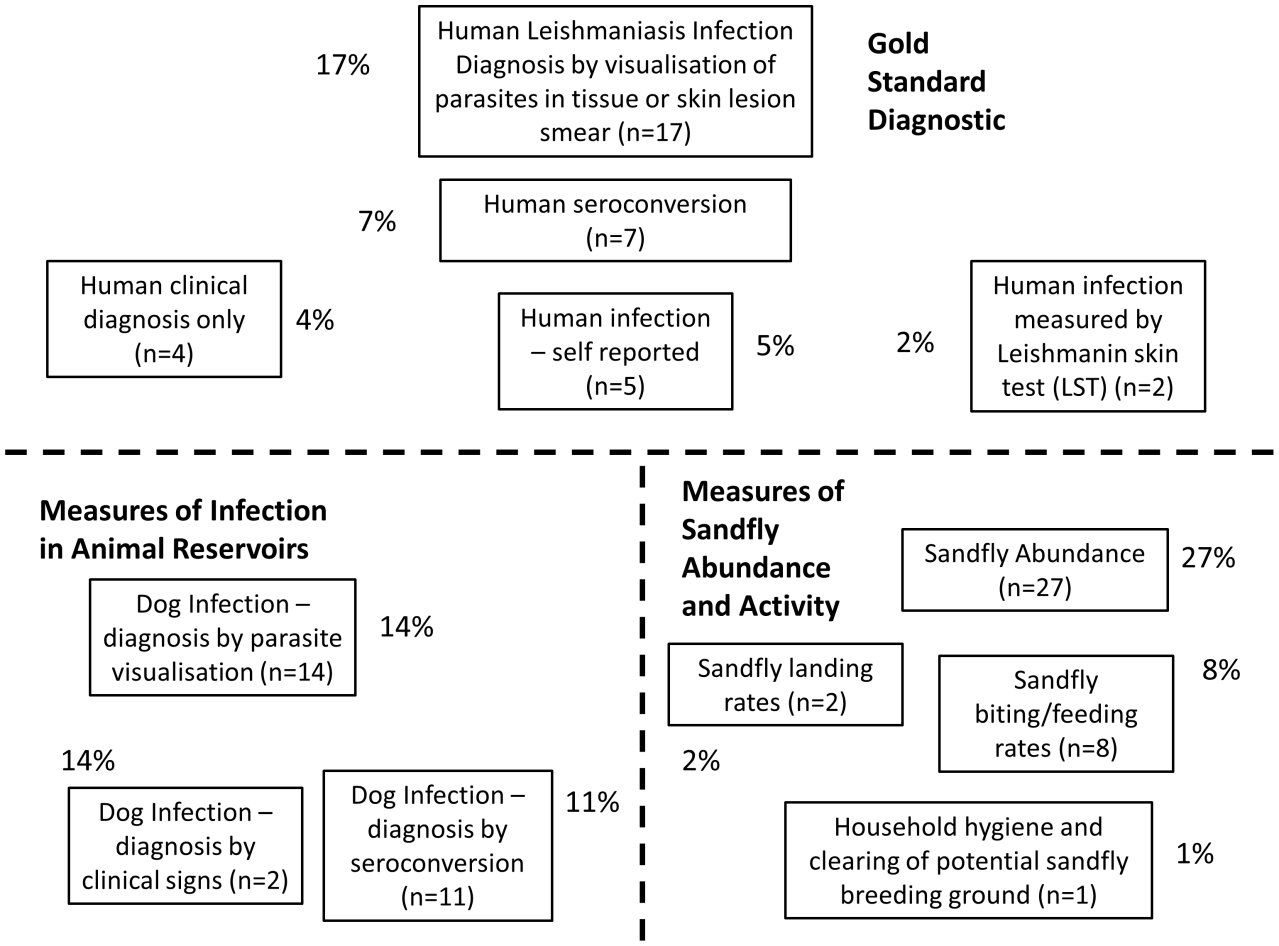
Range of outcome measures identified in review. Number and percentage of total outcome measures. Studies investigating 12 outcome measures were included in this review. These studies were broadly classified into four groups of intervention types, relating to animal reservoir control, vector population control, human reservoir control and concurrently implemented multiple interventions.

In only 35% of studies did the outcome measure involve an assessment of *Leishmania* infection in humans (only these studies with human outcome will be discussed here; however details on all 84 studies are available in the supplemental material section Datasets S1, S2, S3, S4). Of those, 49% (n = 17; 17% of total measurements) used the gold standard of visualisation of parasites in human tissue biopsy or smear in order to confirm diagnosis of infection. Another 20% (n = 7; 7% of total outcomes) used seropositivity as the measure of human infection. The remaining 31% (n = 11; 11% of total) used Leishmanin or Montenegro skin test (LST/MST), clinical signs, or self-reporting of unusual skin lesions by patients, to confirm human infection. The remaining studies use outcome measures such as infection in dogs or measures relating to actual or potential sandfly abundance and animal or human/sandfly interaction (such as landing, biting or feeding rates). Figure 3 shows the number and percentage of total outcome measures graphically.

Before discussing individual categories of interventions, there are some important over-arching points which apply to many studies included in this review.

Firstly, few studies actually measure the outcome of interest i.e. human infection. Of those that do measure human infection, only 49% do so using the gold standard diagnostic technique of visualisation of parasites in smear or biopsy. Twenty percent measure the presence of *Leishmania* antibodies by way of serological tests. Although seroconversion is being increasingly used as a cheap and reliable method of large-scale diagnosis of *Leishmania* infection, it identifies asymptomatic as well as cured individuals, who do not, and might not ever, present with clinical infection. The other outcome measures investigated are intermediate measures used as surrogates for risk of human disease, which may or may not have relevance to human infection.

The second over-arching issue is the lack of generalizability of studies which investigate animal reservoir interventions (in areas of presumed zoonotic transmission) to geographic locations in which transmission is thought to be driven by infection within a human reservoir (anthroponotic transmission).

VL, evidenced by its nomenclature, is the disease resulting from presence of parasites in target viscera: liver, spleen and bone marrow. In India (where the majority of VL is caused by *L.donovani*) it was shown that parasites could be found readily in human blood smears and are thus accessible to the sandfly [19]. Transmission was therefore assumed to be human to human (via sandflies; anthroponotic). However, this was not the case in other areas affected by *L.infantum* (such as the Americas and the Mediterranean) where parasites are not readily found in blood smears [20]. It was hypothesised therefore that there must be another reservoir from which sandflies could become infected and that man was the biological terminal for the parasite [21]. Dogs were incriminated since the first report of canine leishmaniasis in 1908 [22], and infectivity of dogs to sandflies has been shown to be anywhere up to 95% depending on the species of sandfly used for xenodiagnoses [23].

Evidence in the literature as to the efficacy of any animal reservoir intervention programmes in areas of ZVL and ZCL is scant and mixed. Only three controlled trials were retrieved which measure human disease as an outcome in response to animal culling campaigns. Uncontrolled studies measuring dog seropositivity have found that levels of seroconversion in dogs remain stable despite culling programmes [24]. One uncontrolled trial which studied both dog and human seropositivity following prophylactic canine vaccination found a decline in human cases of VL [25]. Notwithstanding the lack of evidence of a link between disease in dog populations and increased human disease, Brazil in particular has spent considerable resources on attempting to control VL transmission through dog culling. Between 1988–1996 the cost of anti-VL interventions in Brazil exceeded $96 million with over 1 million houses sprayed with insecticide and over 150,000 seropositive dogs destroyed [26]. There is no evidence that human mortality and morbidity due to VL has decreased in the region during this time [27].

Incontrovertible evidence of the zoonotic nature of transmission in areas historically considered to be affected by dog-to-human transmission is lacking in the literature. Since not all infected dogs become infectious, the only true way of determining the possibility of a link between canine infection and infectiousness to humans is by xenodiagnosis using uninfected sandflies and subsequent infection of humans [1]. Even though evidence suggests that sandfly vectors are capable of taking up parasites from a blood meal from a dog [23], it is not proof that transmission then occurs to humans. In any event, it is not a suitable diagnostic method for large scale investigation, and may even be unethical to test.

The third major point to consider in studies investigating interventions aimed at reducing the burden of disease in humans is the required sample sizes in order to reliably detect a real difference between the intervention and control groups. For example, whereas 1–3 million malaria deaths and 1–5 billion clinical febrile episodes occur annually in malaria-endemic areas [8], the number of leishmaniasis cases are much smaller; 0.2–0.4 million cases of VL are estimated to occur worldwide every year [7], with only 58,000 cases officially reported. Because of this, the scale of the studies needs to be much larger than for some other vector-borne parasitic diseases. Of the studies using human infection as the outcome measurement, relatively few are sufficiently large to report a statistically significant difference between intervention and control arms.

Within the context of this review, no attempt was made to estimate summary effect measures of specific interventions across multiple studies, because of the degree of heterogeneity of the research. Interventions differed, even within broad categories of intervention type (for example, insecticides varied from study to study; the hole diameter of bed nets differed; vaccines were of different types). Outcome measures also varied. Finally, studies were conducted in many different geographical locations with widely different demographic characteristics of the populations, affected by different species of *Leishmania*, with different potential routes of transmission (thus limiting the generalizability of any findings). The value or appropriateness of single summary measures in such situations is questionable and a decision was made not to produce any.

In view of the importance of measuring outcomes in humans, rather than surrogate measures, which may or may not have relevance to human disease, further discussion is limited to those studies that assessed human *Leishmania* infection following intervention (details of all studies, relating to all outcomes, are available in the supplementary material Datasets S1, S2, S3, S4). The human-specific outcome studies are addressed in the individual category discussions which follow. Those using the most robust diagnostic methodologies (visualisation of parasites through smear of tissue biopsy) will be discussed first, followed by a discussion of the impact of studies using other, less robust diagnostic methods such as serology and skin tests. Tables 1–5 show summaries of studies with human outcome measurements.

**Table 1:** Summary table of human outcome measurements for animal reservoir control interventions (with references in brackets).

ANIMAL RESERVOIR CONTROL	Intervention effective	Intervention ineffective
**Animal culling**		
- Parasite visualisation	[28]	
- Serology		[30]
- Clinical signs	[31]	
**Insecticide-impregnated dog collars**		
- Serology	[29]	
**TOTAL FOR ANIMAL RESERVOIR CONTROL**	**3 studies**	**1 study**

**Table 2:** Summary table of human outcome measurements for vector population control interventions.

VECTOR POPULATION CONTROL	Intervention effective	Intervention ineffective
**Insecticide spraying**		
- Serology		[32], [33]
- Clinical signs	[34]	
- Self reported with check of clinical signs	[35]	
**TOTAL FOR VECTOR POPULATION CONTROL**	**2 studies**	**2 studies**

**Table 3:** Summary table of human outcome measurements for human reservoir control interventions.

HUMAN RESERVOIR CONTROL	Intervention effective	Intervention ineffective
**Treated and untreated nets**		
- Parasite visualisation		[42]
- Serology		[43]
- LST	[46]	
- Self reported	[45], [47], [62]	
- Self reported with check of clinical signs	[35]	
**Insecticide impregnated curtains**		
- Self reported	[52]	
**Insecticide impregnated clothing**		
- Parasite visualisation	[50]	[51]
**Insecticide impregnated bed sheets**		
- Self reported with check of clinical signs	[35]	
**Human vaccines**		
- Parasite visualisation	[56], [63]–[65]	[36], [54], [57], [66]–[69]
**TOTAL FOR HUMAN RESERVOIR CONTROL**	**12 studies**	**10 studies**

**Table 4:** Summary table of human outcome measurements for multiple interventions.

MULTIPLE INTERVENTIONS	Intervention effective	Intervention ineffective
- Parasite visualization (combined effect of impregnated bed nets and curtains and education)	[41]	
- Serology (both studies investigated the combined effect of residual spraying and dog culling)	[33]	[32]
- Clinical signs (concurrent use of deltamethrin-impregnated bed nets, use of insect repellents in the surrounding forest, white washing tree trunks and community-wide education)		[58]
**TOTAL FOR MULTIPLE INTERVENTIONS**	**2 studies**	**2 studies**

**Table 5:** Summary table of all preventative interventions.

ALL PREVENTATIVE INTERVENTION STRATEGIES	Intervention effective	Intervention ineffective
**TOTAL FOR ALL CATEGORIES**	**18 studies**	**14 studies**

### Animal Reservoir Control

This section includes animal elimination, canine vaccines, use of insecticides on dogs; including insecticide-impregnated dog collars, spot-on insecticides (drops applied to skin underneath hair on neck of dog), as well as whole-body insecticide use.

A total of 34 studies were retrieved, of which;

- Four investigated animal elimination
- Eight investigated insecticide-impregnated dog collars
- Six used spot-on insecticide treatments
- One used whole body insecticide use
- Seventeen investigated canine vaccines

Only four of these studies investigated human-specific outcomes as a result of interventions directed at animal reservoir control. Table 1 provides a summary of human outcomes for animal reservoir control studies.

One rodent culling intervention in Iran measured human CL by parasitological confirmation of skin lesion smear [28]. One insecticide-impregnated dog collar study investigated child VL seroconversion using DAT in Iran [29]. One dog culling intervention in Brazil studied human VL seropositivity using ELISA [30], and another dog culling intervention identified paediatric VL cases by passive case detection of clinical disease using health records in Brazil [31].

Three of the four studies reported statistically significant reductions in human *Leishmania* infection between the intervention and control areas. Two of these were animal elimination programmes, and one was an insecticide-impregnated dog collar study.

The only study to measure human infection using the gold standard of parasite visualisation was that of Ershadi et al. [28] who used poisoned grain to eliminate the rodent population around one intervention village and compared human infection to one control village where rodents were not eliminated. This study suffers from lack of generalizability not only to areas of anthroponotic transmission but also to areas of zoonotic transmission where transmission is believed to be driven by dogs. As with all the animal culling studies reviewed here, as well as all but one insecticide study, the report makes no reference to randomisation of areas. Ershadi et al. do however state that pre-intervention rates of infection were similar between the two areas investigated, but no data to support this assertion were presented. Pre-intervention numbers of active rodent burrows vary between intervention and control areas (between two and 18 times more burrows were reported in the control area than in the intervention areas).

Dietze et al. [30] reported that dog elimination in two valleys in Brazil did not result in a significant difference in human seropositivity as measured by ELISA when compared to one control valley. Although the major mode of transmission in Brazil is thought to be zoonotic (with dogs as the major reservoir), the authors hypothesised a greater than previously thought role for humans as the significant reservoir for VL, adding to the uncertainty in the literature that dogs are the most important driver of transmission in areas historically considered zoonotic. The group used active case detection by serology census of humans, using ELISA at six and 12 months following culling of all seropositive dogs in two intervention areas and following no intervention in one control area. The authors did not specify if the eliminated dogs were domestic or feral and no reference to randomisation of intervention and control areas is made. Absence of data on initial numbers of dogs in each valley and how many were eliminated makes it difficult to know if the areas are comparable. If only domestic animals were studied, this would leave a large section of the feral dog population unaccounted for and if the feral population was included, this would make follow-up even more difficult.

Ashford et al. [31] studied paediatric VL cases using passive case detection of clinical disease using health records following dog elimination. The authors used the measurement of cases per 1000 inhabitants, however the actual numbers of inhabitants (and therefore the actual numbers of cases) in each of the two neighbourhoods studied are not stated. In the four years of follow up, the authors reported nine cases per 1000 in the intervention area compared to 35 per 1000 in the control area. As with all studies using passive case detection, potential bias is introduced if one area is systematically better or worse at detecting and reporting cases. This may be linked to other factors such as educational level and socio-economic status of the population studied as well as quality and accessibility of healthcare. There is also a possibility, as with any intervention study, that increased activity focussing on preventative methods may influence the population to take other precautions against acquisition of the disease. None of the studies included in this section reported these kinds of population data. Interestingly, the decrease in incidence of dog infection, as measured by seroconversion, did not differ significantly between the two groups thus adding to the lack of clarity regarding the relationship between canine and human *Leishmania* infection. Like Dietze et al. [30], Ashford et al. [31] do not specify if the elimination programme included domestic and/or feral dog populations.

One of the major problems faced by groups carrying out any kind of animal elimination programme is the lack of control over the numbers of animals actually present in an area. In the case of the rodent elimination study, Ershadi et al. [28] used poisoned grain around rodent burrows. Active burrows were counted and treated every six months for three years if the number of re-opened burrows was 30% or more of initial numbers. Only once in three years did the researchers not need to re-bait burrows with poison meaning that burrows were re-opened rapidly and that the population of rodents may have attained original numbers quite quickly post-intervention. The same is seen of the dog elimination studies by Ashford et al. [31] and Diezte et al. [30] where follow-up of dogs was compromised by virtue of the dog population being both poorly understood through lack of regular censuses, and being dynamic with variable births/deaths/inward and outward movement. It is not clear from either dog culling study whether the elimination included all dogs or just domestic animals.

The only other study in this section which investigated human infection was an insecticide-impregnated dog collar study by Gavgani et al [29] which used DAT to detect seroconversion in children. The group used a matched-cluster randomised trial of 18 villages paired on pre-intervention child VL prevalence. Collars were only fitted to domestic dogs, however the authors note that due to elimination of stray dogs being a generic disease control practise in Iran, the feral dog population is very small and was not considered an issue. Although the intervention is associated with a statistically significant decrease in child VL prevalence during the one year follow up, the actual numbers of seroconversions are low – 17 in the nine intervention villages, and 26 in the nine control villages, and because of this the authors recommend caution be used when interpreting the results of the study.

The lack of studies measuring human infection following intervention resulted in only four out of 34 studies being included in this part of the discussion. Three of those reported a positive effect of the intervention they studied and one reported no statistically significant difference in the intervention and control group. Because of the small number of studies and the potential for bias, limited conclusions can be drawn as to the efficacy of interventions aimed at reducing animal reservoir infection with *Leishmania* on reducing disease burden in humans. In order to address this gap in knowledge, more and larger studies investigating human infection are needed. Ideally these would be cluster randomised in order to attempt to account for known and unknown confounders. Although the fundamental aim would be to reduce the burden of human disease, a secondary outcome would be to clarify whether the canine population is actually driving *Leishmania* transmission in areas historically described as zoonotic, or whether the human reservoir is more important. Without this evidence, it is not possible to determine if there is any use in allocating resources towards controlling animal reservoir populations.

### Vector Population Control

The search for vector population control interventions identified studies relating to indoor and area-wide insecticide spraying, and a range of different interventions broadly termed here ‘Environmental Management’.

A total of 22 studies were retrieved, of which;

- Sixteen studied insecticide spraying of houses, outbuildings and area-wide spraying
- Four studied plastering of walls of houses with mud and/or lime to prevent entry of sandflies
- One investigated use of insecticide on termite mounds and animal burrows
- One measured the efficacy of educating school children on environmental risk factors

Of the 22 studies included in this section, only four investigated human-specific outcome measures following interventions directed at controlling the vector population. All of these involved insecticide spraying of houses and other buildings. Table 2 provides a summary of human outcomes for vector population control studies.

Two studies in Brazil measured seroconversion by ELISA; one measured VL in children under 12 years of age [32] whilst the other included all age groups [33]. One study in the Peruvian Amazon measured human CL by clinical signs [34], and one measured human CL by self-reporting in Afghanistan [35]. Two of the four studies reported statistically significant reductions in human *Leishmania* infection between the intervention and control areas, and two did not.

As a general criticism of studies in this category, only four measured human outcomes, and none studied human infection by parasite visualisation. Only three studies used a cluster randomised study design, with the majority not randomising study areas at all.

Both studies using the most robust diagnostic method (serology) reported no significant difference between the control and intervention groups.

Souza et al. [32] studied VL in children under 12 years in three control and three intervention areas subject to intradomiciliary pyrethroid spraying every six months. The control group is described by the authors as ‘normal activity’ - however this is not defined, nor is it specified if the study area used any preventative interventions as part of their ‘normal’ activity. The researchers needed parental consent to enrol in the study; it is not reported if there was any systematic difference between families who allowed their children to be enrolled, compared to those who did not, or whether there were differences in enrolment between intervention and control areas. Children were tested serologically for leishmaniasis using ELISA every 12 months for 2 years. Two different pyrethroid insecticides are used but there is no mention of which were used when and where. No reference is made to randomization of areas although the authors do state that background infection rates were approximately equal in all three areas.

Nery Costa et al. [33] used random allocation of 34×200 m^2^ areas into four interventions (see also multiple interventions section) in order to study VL in the population living in each area. The intervention area used an unspecified insecticide applied to the inside walls of houses and animal pens whereas the control area used an unspecified insecticide applied to the inside walls of houses only. Background VL prevalence was 42% in the intervention areas and 31% in the control area. It is not clear whether this initial difference was accounted for in the final analysis. Of the 213 seronegative individuals included in the study (authors do not specify how many individuals were in each group), 120 (56%) were followed up during the 6 month study. No indication of drop-out rates by intervention is given and so potential bias could be introduced if the majority of drop-outs occurred in one particular group.

Davies et al. [34] used semi-randomisation (some were randomised, some were matched based on pre-intervention measures) of houses in 3 villages allocated to either intervention (sprayed) or control (unsprayed) arms. Spraying did not occur at the same time for all areas (one village was first sprayed one year later than the other two). Houses allocated to the intervention group were sprayed at six-monthly intervals on four occasions for two villages and only three occasions for the third village. No explanation for differences in randomisation or spraying regimens was given. Cases were detected by active case finding with clinical diagnosis of active lesions. The group reported a significant difference in CL cases over two years between the control houses and the intervention areas (24 versus nine respectively); the differences were more pronounced when cases detected within three months and then six months of the start of the study were excluded . The authors' rationale for removing cases diagnosed within three and six months is that cases within the first six months may reflect infection events which occurred prior to the commencement of the intervention programme. No evidence of existence of a period of time between infection occurring and a positive serology result (pre-patent period) is referenced by the authors. Such *post hoc* analytical decisions may introduce bias. There is evidence in the literature of varied infection rates over time in the same area [36] and epidemics occurring in certain years [37]. Therefore removing data without evidence of a significant pre-patent period of infection, as well as starting the interventions at different times for data that would later be pooled, may not have been appropriate.

One general criticism relating to many of the studies included in this section is that reports exist of interventions (especially residual insecticide spraying) having the effect of directing sandflies into non-sprayed areas [38]. This could be a particular issue when the unit of study is a room or a house, or where areas allocated to intervention and control are directly adjacent. Sandflies are thought to be able to travel anywhere up to 960 m in 36 hours [39]. Therefore, adjacent areas may be more at risk of increased sandfly abundance, and therefore risk of infection, if an intervention has a repellent, but not harmful, effect on sandfly activity.

Finally, Reyburn et al. [35] conducted a large (3666 participants) cluster randomised trial of management of CL in Afghanistan, with four arms (see also multiple interventions section). The sample size was chosen based on power calculations (which none of the other studies reported) and follow-up house visits were conducted at eight, 10 and 15 months post-intervention. The units studied were blocks of 10 houses. Like Nery Costa et al. ^17^, Reyburn et al., also included a safeguard against interventions overlapping into adjacent blocks by including ‘reservoir’ (untreated and not included in the study) blocks. All self-reported lesions were visually inspected before being reported as a case. The authors do state that the intention to confirm diagnosis by smear was not possible due to deterioration of the security situation in Kabul.

All survey workers were blinded to the intervention, and a census was conducted prior to the programme to ensure areas were matched for pre-intervention prevalence. Intervention houses were sprayed only once during the 15 month study period and control and reservoir houses were also offered insecticide spraying of the living areas of their houses although the authors do not report how many houses accepted the intervention. This fact calls into question the validity of the control areas to which the intervention is being compared, however the authors report that since the concentration of the insecticide applied to control houses was so low, it likely did not have an effect on results. The group added that if sandflies had been diverted from intervention to control houses then the protective effect of spraying could have been over-estimated. A loss to follow-up of approximately 45% was reported due to the security situation, however possible bias introduced by this was not considered.

Although Davies et al., [34] do not specify that case detection was self-reported, it is difficult with CL diagnosis that relies on clinical signs, to be certain that all cases have been reported. Reyburn et al., [35] rely on household questionnaire and follow up of clinical signs. Ultimately if someone wanted to hide the fact that they had a skin lesion and the lesion was not on an exposed part of the body, the information could be concealed from researchers, even when active case detection was employed. None of the papers included in this section, or indeed in this review, address the fact that CL diagnosis, especially because of the social stigma lesions can bring to the sufferer [13], could be particularly susceptible to bias in case finding, in comparison to population-wide serological diagnostics.

In summary, only four studies measure human outcome following insecticide spraying of houses and buildings. Two of these use robust diagnostic methodologies and show no difference between intervention and control, and two use less robust methods of diagnosis and show a statistically significant effect. None of the studies measuring environmental management study human-specific outcomes.

Based on this evidence, there is not enough information to determine if insecticide spraying is advantageous in reducing the burden of disease in humans.

In terms of generalizability, indoor insecticide spraying programmes are only useful where the sandfly is likely to come into contact with the walls that are actually sprayed, which necessitates endophagic or endophilic (either biting or resting indoors) sandfly species. It is not clear from any of the papers reviewed here that the authors took this into consideration.

Further research would be needed in order to characterise the feeding, resting and breeding habits of different sandfly species in each endemic area, prior to implementation of a preventative intervention. In addition, more studies are needed which measure human infection after vector control interventions.

Finally, there is evidence in the literature that sandfly resistance to insecticides is emerging, especially to DDT [4] which is still used in large-scale spraying programmes in India [40]. There is also evidence that spraying campaigns are more effective directly before transmission seasons which vary depending on location [40]. Care needs to be taken to ensure that spraying programmes are thoroughly investigated with respect to transmission seasons and sandfly activity in order to have the highest likelihood of being effective. Insecticide spraying is still used during leishmaniasis epidemics [37] and misuse may result in wide-spread resistance which would render them useless when they are needed most.

### Human Reservoir Control

This section includes studies describing personal protection measures for humans such as treated and untreated bed nets, barrier nets, insecticide-impregnated fabrics such as curtains, clothing and bed sheets, and use of soap containing insecticide. It also includes studies on human vaccines. Table 3 provides a summary of human outcomes for human reservoir control studies.

Based on the search criteria, a total of 34 studies were retrieved and included in this section. Five studies which investigated insecticide-impregnated bed nets or other fabrics have multiple arms in order to examine multiple interventions, often including non-impregnated nets and fabrics as a control group. Because of this, there are more interventions than studies. One study [41] investigated the concomitant use of insecticide-impregnated bed nets and curtains and so will be included in the multiple interventions section.

- Sixteen studies investigated insecticide treated bed nets
- Seven examined untreated bed nets
- One study used large area-wide insecticide-impregnated barrier nets
- Two reported on insecticide-impregnated clothing
- Two investigated insecticide-impregnated curtains
- One examined insecticide-impregnated bed sheets
- One study used an insecticide-containing soap
- Eleven studied human vaccines

#### Nets

A total of seven net studies measured a human-specific outcome, however only two of these ([42], [43]) measured human *Leishmania* infection using the most robust methods of parasite visualisation and serology (Table 3).

Both Jalouk et al. [42] and Picado et al. [43] used deltamethrin-impregnated bed nets with 156 holes per square inch. Jalouk et al., used untreated nets as the control group whereas Picado et al. used any existing interventions utilized by the population as the control group. Neither group reported any difference in cases of CL [42] or VL [43] between the treated nets and either untreated nets or existing intervention.

Picado et al. used a paired (based on pre-intervention VL prevalence) cluster randomised trial of 13 intervention and 13 control clusters, enrolling a total of 12,691 individuals. Information on existing interventions used during the 24 month follow up was collected and taken into consideration in the analysis (consisting of irregular spraying of DDT and use of both treated and untreated nets). Loss to follow up was equal between the two groups. Picado et al. concluded that deltamethrin-treated nets do not confer any protection against VL seroconversion. They postulated that in India and Nepal, VL transmission might be occurring outside households as was seen by another treated bed net study measuring indoor sandfly densities [44].

Jalouk et al., also used a paired (based on pre-intervention VL prevalence) cluster randomised trial using five intervention and five control areas, with a total of 10,354 participants. Baseline prevalence was variable (ranging from 4.4% to 20.9%) and increases in CL cases in the untreated and treated areas were not significant.

The net size used in both of these studies was 156 holes per square inch, the size commonly used for malaria prevention. Being approximately one third of the size of mosquitos [4], sandflies are not physically impeded by holes of this size. They do however come into close contact with any insecticide used to impregnate the nets. Nets with smaller holes which physically prevent sandfly entry also limit airflow and are not popular in hot climates [45].

Based on the lack of efficacy of both of these large, paired cluster randomised studies, further research is needed to determine if currently held beliefs regarding the endophagic (indoor feeding) nature of sandflies is true, and whether this varies depending on geographic location, climate or season. If sandflies are endophagic then it would implicate lack of desired efficacy of insecticide, however if not, the sandfly vector would not need to come into contact with the insecticide on the bed net in order to bite humans and transmit *Leishmania* parasites.

The remaining five net studies all report a statistically significant reduction of cases of CL by using insecticide-treated nets. A general criticism regarding these five is that, apart from Tayeh et al., [46] who measured human infection using the Leishmanin skin test (LST), the others all used self-reporting as the method of diagnosis which is at high risk of reporting bias.

Although the studies were large (containing between 2,414 and 10,468 individuals) and therefore better able to detect a difference between intervention and control areas, they are not directly comparable with each other as they were controlled using different interventions (either untreated nets or no intervention), and the bed nets used were of varying hole size (between 50 holes per square inch [47] and 1000 holes per square inch [45]).

Bed net studies have been used extensively in malaria prevention programmes to great effect [48] and the robust cluster randomised study designs appear to have been largely well replicated in leishmaniasis studies. However, as evidenced by the highly heterogeneous studies reviewed here, there are inconsistencies and lack of standardisation in study design which has resulted in groups reporting data which cannot be combined to produce reliable summary estimates.

Being one of the cheapest and most accessible methods of prevention for the general population, bed nets have been shown to be cost effective in the case of malaria [49] and, if effective in prevention of leishmaniasis, would be generalizable to all endemic areas where sandflies bite when people are sleeping.

#### Insecticide-impregnated fabrics

Four studies used insecticide-impregnated fabrics in an attempt to reduce the burden of disease caused by leishmaniasis. Of these, three reported a statistically significant decrease in numbers of cases of *Leishmania* infection between intervention and control groups, and one did not (Table 3).

Both Soto et al. [50] and Asilian et al. [51] investigated the use of permethrin-impregnated clothing for soldiers in Colombia and Iran respectively using a combination of serology and parasite visualisation to confirm diagnosis. Although both study's intervention groups saw less infection, only Soto et al., were able to report a statistically significant decrease in cases of CL in the intervention group compared to the control. Both groups used a double-blind randomised study design. Soto et al., report the area of the bite and noted that one of the cases in the intervention group occurred on the back, which would have been covered “if the subject had followed instructions”. Although the authors do not explicitly state, it seems that there may have been a problem with adherence to wearing impregnated clothing by the soldiers which, if associated with side effects of the insecticide itself (minor skin irritation is reported by both groups), this could introduce bias.

Kroeger et al. [52] investigated the use of impregnated and non-impregnated curtains placed on windows by way of a paired randomised trial design in 569 houses in Venezuela. The study design was informed by pre-intervention prevalence of CL. The group investigated the socio-economic status, occupation and sleeping patterns of the 2913 participants and were able to characterise the feeding patterns of the major sandfly vector in the area (*Lutzomyia* species). This information enabled the authors to identify that sandflies entered the houses through open windows and doors during the evening and that transmission was likely to occur in a domestic setting rather than outside, based on occupational information. Kroeger et al. [52] reported no cases of leishmaniasis in the 1,294 individuals in the intervention groups and 85 cases (8%) in the 1,103 individuals in the control groups. Although the groups used self-reporting as the measure of infection they were blinded as to whether the curtains they received were impregnated or not. Therefore there is no obvious reason for systematic reporting bias to be implicated.

Reyburn et al. [35] conducted a large cluster randomised trial which is discussed in the vector population control section. Briefly, despite the self-reported method of diagnosis and the 45% drop out rate, the group found a statistically significant reduction in cases of CL in areas in which permethrin-treated bed sheets (chaddars) were used. The group does report that chaddars were the least popular of the three different intervention arms investigated in this study (Reyburn et al., also studied spraying and treated bed nets) but do not report on individual adherence to intervention or side effects. If skin irritation did occur as was reported by Soto et al. [50] and Asilian et al. [51] this could have had the effect of reducing usage of bed sheets which would indicate that the already statistically significant protective effect was underestimated.

In summary, the evidence suggests that use of insecticide-impregnated fabric, whether curtains or fabrics worn next to the skin is associated with a decrease in *Leishmania* infection. The generalizability of curtain use would depend on the feeding characteristics of the sandfly vector specific to the area of interest and may only have an effect in areas where transmission occurs indoors. Whereas the side effects of using impregnated curtains were minimal [52], the uptake of using insecticide-impregnated fabrics next to the skin might be problematic if those insecticides cause skin irritation. An important factor in the usefulness of any preventative intervention, which is largely ignored by the studies included in this review, is the implication of affordability and practicality of the intervention. Preventative methods which require individuals to soak clothes in insecticides, which are also skin irritants, on a regular basis might not be practical or acceptable.

#### Human vaccines

All eleven human vaccine studies measured human infection by parasite visualisation. Table 3 shows that four of the studies report a decrease in infection in vaccinated groups, compared to control, whereas seven found no difference. All studies use first generation vaccines (fractions of the parasite or whole killed *Leishmania* with or without adjuvants [53]).

Being human studies, these trials would have been subject to stringent ethical standards perhaps resulting in generally higher quality research than is seen in any other section of this review.

A general problem of human vaccine studies is that after receiving the vaccine, the participants can only be subject to natural transmission of *Leishmania* for obvious ethical reasons. Infection by natural transmission is not guaranteed, and since transmission rates can vary in the same area (evidenced by the fact that Mayrink et al. [36] did not report any cases of CL in three years of observations for either vaccinated or control individuals in an endemic area of Brazil in which there had been consistent transmission in prior years), results may be affected by natural variation of transmission rates.

Once vaccination had been administered, study participants were tested for immunogenicity using LST or MST conversion with the skin test being used as a surrogate marker for protective cell mediated immunity [54]. A positive response is generally thought to be lifelong, but it can revert to negative over time [6]. It is not known if a positive skin test result acquired through vaccine, asymptomatic infection or cured infection, results in life-long immunity. The fact that relapses occur in both VL and CL [55] indicates that life-long immunity is not guaranteed, however it is not clear if susceptibility to relapse occurs after a reversion to a negative skin test. Mayrink et al. [56] note that the members of the vaccinated group who became infected were also correlated with poor skin test reactivity, whereas Armijos et al. [57] found equal numbers of parasitologically confirmed CL infections in vaccine and control groups, even when LST conversion was 74% in the vaccinated group compared to 15% in the control group.

In summary, the studies show that producing a reliable human vaccine is complex. It is not clear whether the currently available skin tests are a useful surrogate for protective immunity; it is also not clear if the positive skin test result induced by vaccination is of equal magnitude or has the same protective effect as a positive result acquired naturally. Any diagnostic value of the skin test may be lost should vaccine use become widespread before these fundamental issues are elucidated.

#### Multiple interventions

Four studies included multiple interventions which were applied to the population of interest concurrently and therefore the separate effect of each specific intervention could not be determined. Table 4 shows a summary of study outcomes.

A general criticism of the multiple intervention studies is that it is not possible to estimate individual effects of interventions as they are all used concurrently. In comparison to studies with multiple arms each containing a small number of individuals, multiple intervention studies do have the advantage of having all individuals in one group, therefore potentially increasing the power of the study.

Moosa-Kazemi et al. [41] studied the use of pyrethroid-impregnated bed nets, window and door curtains, and health education (relating to leishmaniasis risk factors and proper use of bed nets and curtains), compared to untreated bed nets, curtains and education, compared to no treatment in a total of 480 households. The group reported a statistically significant decrease in parasitologically confirmed cases of CL in one year, in households using the insecticide-treated bed nets and curtains in three randomly selected neighbourhoods in Iran which were matched for pre-intervention prevalence of CL. The group used bed nets with 150 holes per square inch which are the nets commonly used in malaria prevention campaigns.

The group did not see a statistically significant effect of using non-insecticide-treated bed nets or curtains on cases of CL compared to the control area, thus suggesting that the protective effect of the treated intervention group was due to the insecticide and not the physical presence of a net. The authors report on bed net usage by households, including what time the bed nets were put up, where they were placed, and if the nets were washed during the study. The results did not show any difference in net usage between the treated and untreated areas.

Both Nery Costa et al. [33] and Souza et al. [32] investigated human VL infection by serological methods following the concurrent spraying of insecticide and dog culling. Nery Costa et al. [33] claim significance whereas Souza et al. [32] do not. Neither study reports P values. The majority of the individual study criticisms are previously discussed in the vector population control section in which both studies also appear and will not be discussed again here due to limited space. One key point to highlight is that the statistically significant decrease in human VL suggested by Nery Costa et al. is called into question by the study design. The group compared human seroprevalence following spraying of houses and elimination of infected dogs with spraying of houses and animal pens, and eliminating infected dogs. The results show that the protective effect conferred by spraying houses and eliminating dogs (Odds Ratio of 0.2 (95% CI 0.04–0.89)) is lost when animal pens are included in the spraying regimen (Odds Ratio of 0.69 (95% CI 0.27–1.76). Given the 44% loss to follow up, interpretation of the findings is difficult.

Rojas et al. [58] used multiple interventions consisting of treated bed nets, modification of sandfly resting areas and health education. CL prevalence was measured by active case finding of clinical signs during one year of follow up. The 20 villages included in the study were matched for CL prevalence, number of inhabitants and level of community participation prior to the start of the study, and then randomised to intervention or control (no intervention). Data on potential confounders, such as age, occupation, proximity of house to forest and building materials used for house construction, were collected and results adjusted for these. Every three months the interventions were re-implemented (bed nets re-impregnated, trees re-white washed etc). The authors specified that individuals with a previous history of CL and who developed active skin lesions during the study were also included as incident cases, although these cases were not included in the data analysis. All other studies investigated in this review did not include data concerning individuals with any history of skin lesion or with a positive skin or serology test. The authors use the justification that re-infection with CL has been documented, and that cases of re-infection should also be targeted by intervention methods. Loss to follow up was reported and was approximately equal in both the intervention and control groups. With 2,738 participants, this study is the largest to be included in this section.

To summarise, the quality of studies varies in this section; however two of the three studies using the most robust diagnostic methods report no statistically significant difference between the control and intervention groups, and the third reports a significant result, but there were large losses to follow up.

### Limitations of Review

Due to the heterogeneity of study designs and outcome measures, a full and formal quality assessment of studies was not carried out. Although the variable methodological quality seen in the studies reviewed here is addressed in this discussion section, this was not carried out systematically. It may be advantageous for future work to systematically rate methodological quality and take this into consideration when reporting results. The authors are aware of one potentially relevant paper published in a Chinese language journal but were unable to obtain the reference.

### Summary and Conclusions

At the time of writing, published protocols highlight the intention to investigate aspects of prevention of *Leishmania* infection in humans [59]–[61]. To date however, there are no published reviews which address preventative methods in their entirety. This review provides a comprehensive overview of all interventions against human leishmaniasis, highlighting fundamental gaps in knowledge, and suggesting directions for future research.

Four broad categories of preventative interventions were identified in this review, investigating a heterogeneous mix of outcome measures and using a variety of different methods.

This review emphasizes the absence of high quality evidence demonstrating the impact of interventions on the prevalence or incidence of human *Leishmania* infection assessed using reliable diagnostic modalities.

Research identified within this review includes intervention strategies ranging from protection of humans against infection, to interventions aimed one stage upstream of human infection (targeting the sandfly vector), and even further, to interventions targeting animal reservoir species.

Conflicting data on the impact of dogs in transmission of leishmaniasis to humans, along with lack of generalizability of interventions directed at vector control, point towards gaps in fundamental knowledge of the biology of transmission. Despite this weak evidence base, many countries continue to invest heavily in preventative methods focussed on control of leishmaniasis in dogs.

Based on our current lack of understanding of the transmission of *Leishmania*, it seems salient to focus scant resources on prevention of human infection, as opposed to interventions which attempt to address upstream risk.

The absence of a promising prophylactic human vaccine candidate, along with the scarcity of human vaccine studies, indicates that an effective (and cost-effective) human vaccine is unlikely to be forthcoming in the near future. Nonetheless, with no reliable intervention having been identified and the current treatment options for leishmaniasis being expensive, with serious side effects and emerging resistance, there is clearly a need for a more integrated focus within the international community to direct resources towards development of a human vaccine.

### Final Conclusions

#### Main areas for immediate research

- More work is needed to develop a cheap, rapid, sensitive and specific diagnostic test which is not hindered by regional variation or the need for specialist equipment. It should also be able to differentiate infective and non-infective individuals irrespective of whether there is clinical evidence of infection.
- More work is needed to determine which species are the main reservoirs of infection in which locations. If human, better treatment options are needed to treat symptomatic and asymptomatic, infective individuals. If animal, cost effective and ethically acceptable methods of control will be needed.

Best methodologies of evaluating controls strategies for leishmaniasis for funding by agencies and countries:

- Studies should measure human specific outcomes as the primary endpoint using appropriate diagnostic modalities.
- Intervention studies need to be fit for purpose for the location in which the intervention might be used. Different species of *Leishmania* display substantial epidemiological variation in patterns of transmission.

Before conducting intervention studies to prevent transmission of leishmaniasis by the sandfly vector, a thorough survey should be conducted to identify:

- Vector-specific habits/behaviours relating to transmission such as location of where sandfly bites occur (indoors or outdoors), sandfly biting seasonality and time of day/night.
- Reservoir-specific habits/behaviours relating to species of animal infected, presentation of symptoms.
- Human at risk population habits and behaviours – acceptability of interventions, types of employment, knowledge of disease and symptoms

## Supporting Information

Dataset S1 Full dataset of Animal reservoir control studies.
(DOCX)

Dataset S2 Full dataset of Vector population control studies.
(DOCX)

Dataset S3 Full dataset of Human reservoir control studies.
(DOCX)

Dataset S4 Full dataset of Multiple interventions studies.
(DOCX)

Text S1 Search strategy for database searching of Medline, EMBASE, CENTRAL, Web of Science, LILACS and WHOLIS.
(DOCX)
