# Supplementary material for: A Review of Preventative Methods against Human Leishmaniasis Infection
Source: PLoS Negl Trop Dis. 2013 Jun 20;7(6):e2278. doi: 10.1371/journal.pntd.0002278 (PMC3688540; doi:10.1371/journal.pntd.0002278)
Supplement: Dataset S1 — Full dataset of Animal reservoir control studies. (DOCX) [file pntd.0002278.s001.docx]

Animal culling studies

| Paper (author, title, year) | Study type | Number of subjects/areas | Location of study | Type of intervention | Method of diagnosis of infection | Primary outcome investigated | Secondary outcomes investigated | Main findings | Secondary findings | Comments | Intervention effective? |
| --- | --- | --- | --- | --- | --- | --- | --- | --- | --- | --- | --- |
| Ershadi, 2005. Rodent control operations against zoonotic cutaneous leishmaniasis in rural Iran. | Non-randomised controlled trial | 2 villages; intervention 300 inhabitants, and control 400 inhabitants | Iran | Rodent control operations by baiting rodent holes using zinc phosphide once a month in May, June, July and September in 1997 within a 500 meter circle of houses in the intervention area. No intervention used in the control areas. | Active case finding by house-to-house visits questioning about active lesions. Those with active lesions had smears taken for verification of diagnosis. | Self-reported prevalence of human zoonotic cutaneous leishmaniasis (ZCL) by presence of active skin lesions. |  | Intervention cases per 1000 vs control: 1998 50.3 vs 191.5, 1999 163.1 vs 297.4, 2000 10.8 vs 208.7, 2001 15.5 vs 109.3, 2002 56.8 vs 410.3. The incidence of ZCL in the intervention group was less than in the control village during 1998-2002. Differences are statistically significant (P<0.005). |  | Active burrows were counted and treated every six months (in the three years) if the number of reopened burrows was 30% or more of initial numbers. Only once in these three years did the researchers not need to re-bait burrows with poison – burrows were reopened rapidly. | Yes |
| Ashford, et al., 1998. Studies on control of visceral leishmaniasis: impact of dog control on canine and human visceral leishmaniasis in Jacobina, Bahia, Brazil. | Non-randomised, Controlled trial | 2 areas: 1 intervention, 1 control area | Jacobina, Bahia, Brazil | Domestic dog culling. Between June 1989 and May 1993. | Paediatric cases identified by passive case detection of clinical disease using health records. Dog cases identified by an annual census of domestic dogs - serologic tests performed using FAST-ELISA. | Human paediatric cases (<15 years) of visceral leishmania |  | Significant decrease in incidence of disease cases in children <15yrs intervention vs control - the 4 years post intervention saw 9 cases per1000 among intervention area compared to 35 per 1000 in control area (P<0.01). | In the four years of intervention, the decrease in incidence in the intervention area was not significantly different from the observed incidence in the control area (P=0.07). | Difficult to measure seroconversion in dogs as the dog population is dynamic with new births/deaths and inward/outward migration. | Yes. |
| Braga, et al., 1998. Controle do calazar canino: comparação dos resultados de um programa de eliminação rápida de cães sororreagentes por ensaio imuno-enzimático com outro de eliminação tardia de cães sororreagentes por teste de imunofluorescência indireta de eluato de papel filtro. (paper in Portuguese language). | Non-randomised controlled trial. | 2 areas: 1 intervention, 1 control area. | North East Brazil, São Luiz do Curú. | Dog Culling - Fast diagnosis with elimination of animals demonstrating infection within 7 days (intervention) or normal practice of elimination within 80 days (control) | Either blood eluate immunofluorescence (Control group) or ELISA (Intervention group). | Seroprevalence in dogs within area of intervention/control. |  | In the control area, a 9% decrease in seroprevalence was noted (the test identified 37% seropositives (93/254) prior to intervention, and identified 28% seropositives (66/239) post intervention. In the intervention area, a 27% decrease in seroprevalence was noted (the test identified 46% (126/276) seropositive dogs pre-intervention, and 19% (38/197) post intervention). The reduction of identified cases in intervention compared to control area is statistically significant (p = 0.0015). |  | Cannot tell from paper if culled dogs were feral, domestic or both. | Yes |
| Dietze, et al, 1997. Effect of Eliminating Seropositive Canines on the Transmission of Visceral Leishmaniasis in Brazil. | Non-randomised controlled trial. | 3 areas - 2 intervention, 1 control. 469 humans and 140 dogs. | Valleys of Pancas, Espırito Santo, Brazil. | Dog culling - All seropositive dogs culled in 2 intervention areas at both study initiation and at 6 months. | Dot-ELISA | Human and dog seropositivity rates measured by active case detection - full census at study initiation, 6 and 12 months of both humans and dogs. |  | During the 12-month study period,  human seropositivity rates, as measured by dot - ELISA, increased from 15% (49/267) to 33% (79/240) at six months and to 54% (113/209) at 12 months post-intervention in the intervention valleys and from 14% (28/202) to 36% (55/151) at six months, and to 54% (80/149) at 12 months post-intervention in the control valley. The elimination of infected canines in the intervention valleys did not result in a statistically significant difference between the incidences of human serological conversion in the intervention and control valleys at either 6 (P=0.5) or 12 months (P=0.87). The role of humans as a significant reservoir for American Visceral Leishmania (AVL) is proposed as an explanation for the study results. |  | The number of dogs participating in the assay in each valley  is not revealed. Cannot tell from paper if culled dogs were feral, domestic or both. | No |

Insecticide use on dogs

| Paper (author, title, year) | Study type | Number of subjects/areas | Location of study | Type of intervention | Method of diagnosis of infection | Primary outcome investigated | Secondary outcomes investigated | Main findings | Secondary findings | Comments | Intervention effective? |
| --- | --- | --- | --- | --- | --- | --- | --- | --- | --- | --- | --- |
| Molina et al, 2012. Efficacy of 65% permethrin applied to dogs as a spot-on against  Phlebotomus perniciosus. | Randomised controlled trial. | 6 treated and 6 untreated control dogs. | Spain. | Permethrin spot-on topical insecticide treatment. Dogs exposed to sandflies once a week for 7 weeks. | NA | Repellent and insecticidal efficacy. |  | The treatment had a statistically significant (P<0.05) repellent and insecticidal effect between treated and control groups until day 36. |  | Although the authors report a statistically significant difference between treated and control groups until day 36 at the P<0.05 level, they report that levels of insecticidal and repellent activity were only acceptable until day 22. No reasoning for a difference in statistical significance and acceptable levels was given by the authors. | Yes, until 22 days post treatment. |
| Courtenay et al, 2009. A long-lasting topical deltamethrin treatment to protect dogs against visceral leishmaniasis. | Controlled trial. | 52 dogs. | Brazil. | Insecticide for dog (whole body wash as a pour-on); emulsifiable concentrate solution or suspension concentrate formula or water (control). | NA | Anti-feeding effect (proportion of female sandflies unfed) |  | Percentage of female flies that were alive and blood fed at 24hr post exposure to either formulation ranged from 1.5% (day0) to 25% (day 134) compared to 85% and 71% respectively in the controls (Odds Ratio 0.16, P<0.0001). |  |  | Yes |
| Aoun et al, 2008. Efficacy of Deltamethrine-impregnated collars Scalibor in the prevention of canine leishmaniasis in the area of Tunis. (Paper in French language). | Controlled intervention. | Eighty leishmaniasis free dogs (42 collared and 38 as control dogs). | Tunisia. | Deltamethrin impregnated dog collars. | ELISA. Infection confirmed by culture of parasites or PCR | Susceptibility of dogs to Leishmania infantum infection - measured by seroconversion. |  | 15.8% (6/38) control dogs were infected by *L.infantum* during the study period against zero in the collar group (P=0.02). |  |  | Yes |
| Ferroglio et al, 2008. Evaluation of 65% Permethrin Spot-on and Deltamethrin-impregnated Collars for Canine Leishmania infantum Infection Prevention. | Non-randomised controlled intervention. | 120 dogs in 65% permethrin group, 119 dogs in deltamethrin collar group and 188 control dogs with no intervention. | Liguria, Italy. | 65% Permethrin Spot-on treatment (applied to skin underneath hair on neck of dog) and Deltamethrin-impregnated Collars. | IFAT | Seroconversion of dogs. |  | 2.5% (3/120) and 2.5% (3/119) in each intervention group and 15% (30/188) in control group. Statistically signiﬁcant difference between these two groups and the control group (P=0.0004). In both permethrin and deltamethrin-treated groups, an 84% reduction of incidence, by serology, was observed compared with the control group. |  |  | Yes |
| Manzillo et al, 2006. Deltamethrin-impregnated collars for the control of canine leishmaniasis: Evaluation of the protective effect and influence on the clinical outcome of Leishmania infection in kennelled stray dogs. | Randomised controlled intervention. | 120 clinically healthy and Leishmania-seronegative dogs (50% in each group - intervention or control). | Campania, Italy. | Deltamethrin-impregnated collars. 2-year field study on kennelled stray dogs. | Serological IFAT. | Seroconversion of dogs. |  | After the 2003 season, cross-sectional serological examinations tested positive in 11.4% (5/44) collared animals and 41.2% (14/34) controls, with 72.3% estimated protection (P < 0.005). After the 2004 season, 22.6% (7/31) seronegative collared dogs seroconverted compared with 41.2% (7/17) seronegative controls (P = 0.15). At the end of the study, the cumulative rate of protection was 50.8% (P = 0.005). | At the clinical evaluation of 21 seroconverted dogs from both groups, canine leishmaniasis signs were significantly more frequent (90% versus 36%, P = 0.017) and rapidly progressive in uncollared than in collared dogs. | Collar losses during the two seasons were high (35%). About 50% of enrolled dogs were lost at follow-up because of death or they were moved to other locations. | Yes |
| Otranto et al, 2007. Efficacy of a combination of 10% imidacloprid/50% permethrin for the prevention of leishmaniasis in kennelled dogs in an endemic area. | Negative-controlled field study | 631 dogs negative for Leishmaniasis - split into two groups and then into 3 different treatments | Southern Italy | Spot on treatment (applied to skin underneath hair on neck of dog). Group A—treated with imidacloprid and permethrin once a month; Group B—treated every 2 weeks; and Group C—untreated control animals. | Serologically tested by rK39 dipstick and lymph-node smear with PCR | Incidence of dog infection (seroconversion) |  | Incidence/100 dog years for both areas combined; Group A: 1.01 (0.96-1.44) (P<0.01), Group B: 0.68 (0.49-0.88) (P<0.01), Group C (Control): 9.80 (9.17-10.55). |  |  | Yes |
| Miro et al, 2007. Repellent Efficacy of a Combination Containing  Imidacloprid and Permethrin against Sand Flies  (Phlebotomus papatasi) on Dogs. | Randomised controlled trial. | 8 treated and 8 untreated control dogs. | Spain. | Imidacloprid/permethrin spot-on topical insecticide treatment. Dogs exposed to sandflies on days 1, 7, 14, 21, and 28 after applying the product. | NA | Repellent and insecticidal  efficacy of imidacloprid/permethrin. |  | The product had an insecticidal efficacy on female sand flies of 53.2% (day 1), 49.4% (day 7), 15.1% (day 14), 13.2% (day 22), and 2.9% (day 29). Only within the first week of application was the insecticidal effect significant (P≤0.05). The product showed a repellent effect of 97.7% (day 1), 96.3% (day 7), 96.5% (day 14), 92.7% (day 22), and 74.0% (day 29). The efficacy of the repellent effect and insecticidal activity differed statistically between treated and control dogs from day 1 onwards (P≤0.05). |  |  | Repellent activity was effective until day 29 however insecticidal activity ceased to be effective after day 7. |
| Gavgani et al, 2002. Effect of insecticide-impregnated dog collars on incidence of zoonotic visceral leishmaniasis in Iranian children: a matched-cluster randomised trial. | Matched-cluster randomised trial. | 18 villages were paired, matched on preintervention child prevalance of *L.infantum* infection. | Iran | Deltamethrin impregnated dog collars fitted to all dogs in the 9 intervention villages, no intervention in the 9 control villages. | DAT | Incidence of child *L.infantum* infection measured by seroconversion. | Seroconversion in dogs. | Seroconversion rate in children was 1.49% (17/1141) in intervention villages and 2.41% (26/1078) in control villages (OR 0.57, 95%CI 0.36-0.90 p=0.017). | The seroconversion rate in dogs in intervention villages was also significantly reduced (0.46, 0.30-0.70, p=0.0003) |  | Yes |
| Giffoni et al, 2002. Evaluation of 65% Permethrin Spot-On for Prevention of Canine Visceral Leishmaniasis Effect on Disease Prevalence and the Vectors (Diptera Psychodidae) in a Hyperendemic Area. | Non-randomised controlled intervention. | 160 control dogs and 230 intervention dogs. | Brazil | 65% permethrin spot-on insecticide (applied to skin underneath hair on neck of dog). | Active case finding in dogs with indirect immunofluorescence assay (IFAT). | Infection in dogs. |  | The infection rate for treated dogs 1 month following the final treatment was approximately 50% reduced from that observed before treatment (19.3% (29/150) vs 9.6% (9/94)). Conversely, the infection rate at the control site was more than 80% higher at the September sampling than that observed pretreatment (4.1% (6/146) vs 7.4% (9/121)). |  | The authors do not specify how many dogs were included from start or were lost from the study. No P values given. | Yes |
| Maroli et al, 2001. Evidence for an impact on the incidence of canine leishmaniasis by the mass use of deltamethrin-impregnated dog collars in southern Italy. | Non-randomised controlled intervention | During two consecutive transmission seasons, collars were fitted to 350 (1998) and 354 (1999) dogs from one area (70% of the canine population). Control dogs (371 and 264 in the 2 years, respectively) were from four towns of the same area. | Campania, Italy. | Deltamethrin-impregnated dog collars. | IFAT. Sometimes diagnosis confirmed with lymph or bone marrow smear. | After each transmission period, incidence rates of seroconversions  were determined in adult dogs that were serologically negative before the season  under evaluation, and in puppies. |  | After the 1998 season, 2.7% (6/244) of the dogs in the  intervention town seroconverted compared to 5.4% (16/317) in the control towns (50%  protection, P = 0.15). After the 1999 season, 3.5% (4/114) of collared dogs seroconverted  compared to 25.8% (24/93) of control dogs (86% protection, P < 0.001). |  |  | Yes |
| David et al, 2001. Deltamethrin-impregnated dog collars have a potent anti-feeding and insecticidal effect on *Lutzomyia longipalpis* and *Lutzomyia migonei*. | Controlled intervention. | 3 dogs wearing Deltamethrin-impregnated PVC dog collars, 3 controls. | Brazil. | Insecticide-impregnated dog collars. | NA | Sandfly feeding rates. | % mortality of sandflies. | Anti-feeding effect was 99.3% (4 weeks after application), 100% after 8 and 12 weeks and 96% at 16 and 20 weeks for *L. longipalpis*. The difference between treated and untreated collars was highly significant at P<0.0005). Mortality ranged from 96% at 4 weeks to 35% at 35 weeks, the difference between treated and untreated collars was significant P<0.0005). | Mortality initially was over 90% and at 35 weeks was 35% (for *L. longipalpis*) and 46% (for *L. migonei*). |  | Yes |
| Reithinger et al, 2001. Topical Insecticide Treatments to Protect Dogs  from Sand Fly Vectors of Leishmaniasis. | Controlled trial. | 17 dogs in total. 14 treated and 3 control. | Brazil. | 5 dogs with Deltamethrin (DM)-impregnated collars, 3 dogs with diazinon-impregnated collars, 3 dogs with permethrin  topical lotion, 3 dogs with fenthion topical  lotion, and 3 untreated negative controls. | NA | Sandfly feeding and mortality. |  | Bloodfeeding rates were significantly  lower on dogs treated with DM, permethrin, and fenthion (compared with untreated dogs) at both 1 month (P<0.001; P=0.010; and P=0.005, respectively) and 2 months (P <0.001; P=0.004; and P=0.018, respectively). No significant reduction in bloodfeeding was detected on diazinon-treated dogs at any time point, when compared with negative control dogs. In comparison with untreated dogs, death of unfed sand flies was significantly increased by 41%, i.e., 4.1-fold (1.7 to 6.6) at 1 month by DM treatment (P=0.004) and by 58%, i.e., 5.4-fold (2.3 to 7.6) by fenthion treatment (P=0.001). The death rate of unfed sand flies at 2 months was significantly increased by 29%, i.e., 2.6-fold (1.02 to 4.7) by  DM treatment (P=0.046). |  | Authors use mortality of both fed and unfed sandflies however protection of dogs from Leishmania infection would only occur if sandfly mortality was high before biting and feeding. | DM, permethrin and fention were all effective at decreasing blood feeding rates and increasing mortality of sandflies at various timepoints, diazinon not effective at any time point. |
| Killick-Kendrick et al, 1997. Protection of dogs from bites of phlebotomine sandflies  by deltamethrin collars for control of canine leishmaniasis. | Controlled trial. | 2 deltamethrin-impregnated collared dogs and 2 control dogs (without collars). | France. | Deltamethrin-impregnated dog collars. | NA | Sandfly mortality and anti-feeding effects. |  | Impregnated collars protected dogs from 96% of the bites (1911 female sandflies were engorged on collar-less dogs compared to 75 on collared dogs between weeks 2 and 34).Overall mortality of sandflies was <16% with control dog5 and >15% with collared  dogs. |  | Authors conclude that intervention was effective at reducing feeding of sandflies on insecticide-impregnated collars however no P values are given. | Yes but no P values reported. |

Canine Vaccine Studies

| Paper (author, title, year) | Study type | Number of subjects/areas | Location of study | Type of intervention | Method of diagnosis of infection | Primary outcome investigated | Secondary outcomes investigated | Main findings | Secondary findings | Comments | Intervention effective? |
| --- | --- | --- | --- | --- | --- | --- | --- | --- | --- | --- | --- |
| Carcelen, 2009. The chimeric multi-component Q protein from Leishmania in the absence of adjuvant protects dogs against an experimental Leishmania infantum infection | Double-blind placebo controlled experiment in dogs. Second generation vaccine*. | 21 healthy (seronegative) Beagle dogs into 3 groups (7 in each group). | Spain | Recombinant protein, named Q formed by the genetic fusion of five intracellular antigenic fragments from *L.infantum.* Group Q received a single Q dose on day 0, Group Q+Q received two Q doses on days 0 and 21, control dogs received placebo. | Serology and clinical symptoms detected by veterinarians, and detection of parasite after necropsy. | Number of dogs with clinical symptoms and presence of parasites after challenge with *L.infantum* promastigotes 60 days after first vaccination. |  | Among the control group, 86% (6/7) dogs developed clinical symptoms compared to 43% (3/7) for the Q group, and 71% (5/7) in the Q+Q group. After parasitological culture analysis, between 57-71% presence of parasites depending on the organ cultured (4/7) spleen, (5/7) lymph node for the Q group, and between 57-86% (4/7) for spleen and (6/7) lymph node for the Q+Q group. The authors conclude that the Q and the Q+Q groups were able to induce parasite clearance or a significant reduction of parasite burden (P<0.0001). |  | Different methods of parasite detection were used – culture, tissue smears and PCR. Slightly different results came from each method. | Yes. |
| Fernandes, 2008. Protective immunity against challenge with Leishmania (Leishmania) chagasi in Beagle dogs vaccinated with recombinant A2 protein. | Randomised controlled trial. Dogs. Second generation vaccine. | 21 dogs (14 intervention vaccine and adjuvant group, 4 control dogs received saline solution and 3 control dogs received adjuvant only). | Brazil. | A2 recombinant protein with saponin adjuvant. 50% (7/14) intervention dogs and all control dogs were challenged with *L.chagasi* promastigotes. | Bone marrow and peripheral blood culture of parasites and clinical symptoms. | Numbers of dogs infected (presence of parasites and clinical signs) after challenge. |  | After 9 months post challenge, 57% (4/7) vaccinated dogs (which were challenged) showed presence of parasites from bone marrow culture and 29% (2/7) showed clinical symptoms compared to 100% (7/7) control dogs which showed presence of parasites in bone marrow and 71% (5/7) which showed clinical signs (which the authors note were much more severe in the control group than the vaccinated group). |  | No P values reported for outcomes. | Yes |
| Moreno, 2007. Immunization with H1, HASPB1 and MML Leishmania proteins in a vaccine trial against experimental canine leishmaniasis. | Randomised blinded controlled trial. Dogs. Second generation vaccine. | 48 seronegative dogs in total – divided into 7 groups (with 8 dogs/vaccine group and 4 per adjuvant group). Group A: HASPB1, Group B: H1, Group C: HASPB1+H1 (adjuvant used for Groups A-C was Montanide), Group D: Montanide, Group E: MML, Group F: MPL-SE, Group G Control (saline injection). | Spain. | 3 Leishmania recombinant proteins – Histone 1 (H1) and hydrophilic acylated surface protein B1 (HASPB1) immunized singly, or together as a protein cocktail vaccine with Montanide and the polyprotein MML immunized with MPL-SE adjuvant. |  | Clinical signs of infection in dogs post intravenous challenge with *L.infantum* promastigotes carried out by a veterinarian blinded to the intervention group. |  | 62.5% (5/8) dogs immunized with H1 Montanide and 50% (4/8) of dogs immunized with either the combination of HASPB1 with Montanide or the combination of H1+HASPB1 with Montanide remained free of clinical signs of infection after challenge with *L.infantum* promastigotes. Compared to 28.7% (2/7) dogs immunized with the polyprotein MML and adjuvant MPL-SE, and 25% (2/8) dogs in the control group which remained free of clinical signs of infection. |  | Authors state that the vaccines containing antigen were able to provide partial protection however numbers were too low to be able to establish statistical significance. | Partial protection. |
| Rodriguez-Cortes, 2007. Vaccination with plasmid DNA encoding KMPII, TRYP, LACK and GP63 does not protect dogs against Leishmania infantum experimental challenge. | Randomised controlled trial. Dogs. Second generation vaccine. | 12 dogs randomly assigned to 2 groups receiving (at 15 day intervals), either 4 doses of plasmid DNA (intervention) or saline (control). | Spain. | Multiantigenic plasmid DNA vaccine encoding KMPII, TRYP, LACK and GP63 *L.infantum* antigens. | Bone marrow and tissue samples taken (after necropsy). Serology by ELISA. | One month after last vaccine dose, dogs were experimentally challenged with *L.infantum* promastigotes. Outcome was to determine parasites (or parasite DNA) in blood and tissues after necropsy. |  | Clinico-pathological findings were similar and no statistically significant differences were observed between groups throughout the follow-up period. All (100%) dogs in the vaccinated group showed Leishmania DNA in blood samples during follow-up. 2 control dogs showed low or no parasitaemia throughout the course of infection. Median parasitaemia in vaccinated dogs was statistically higher than in control dogs at months 9 and 10 p.i (P=0.041). Leishmania DNA at necropsy was detected in liver, spleen and lymph node of both control and vaccinated dogs (no statistically significant difference.) |  | Clinical symptoms were reported for all dogs however some were only transient and disappeared over time. | No |
| Lemesre, 2007. Long-lasting protection against canine visceral leishmaniasis using the LiES Ap-MDP vaccine in endemic areas of France: double-blind randomised efficacy field trial. | Double-blind randomised efficacy field trial. Dogs. Second generation vaccine. | 414 seronegative dogs, 205 vaccinees and 209 placebo-treated control animals. | France. | LiES Ap (naturally excreted/secreted antigens, purified from culture supernatant of *L.infantum* promastigotes) with muramyl dipeptide (MDP) adjuvant. 82% (340/414) (175 controls and 165 vaccinees) were analysed for clinical, serological and parasitological studies at 24 months post-vaccination, after two sandfly seasons. | Bone marrow culture of parasites. | Number of dogs infected after natural challenge. |  | After 2 year follow up, Leishmania infection rate was 0.6% (1.165) in vaccinated dogs and 6.9% (12/175) in the placebo group. The efficacy of the vaccine was calculated to be 92% (P=0.002). |  | Natural challenge does not guarantee that all dogs will be exposed to infection. Healthy seronegative dogs were admitted to the study even though they were PCR positive at the bone marrow examination. No explanation was given for this. Sixty-six dogs (29 placebos and 37 vaccinees, 15.9%) were eliminated from the study due to non-study related death or disappearance. | Yes |
| Lemesre, 2005. Protection against experimental visceral leishmaniasis infection in dogs immunized with purified excreted antigens of Leishmania infantum promastigotes (LiES Ap). | Controlled clinical trial with 4 arms. Second generation vaccine. | 18 healthy (seronegative) Beagle dogs were allocated into four groups). | France. | Received at 3-week interval either two subcutaneous injections of 50μg (group 2 n=3), 100μg (group 3 n=6) or 200μg (group 4 n=3) LiES Ap in formulation with muramyl dipeptide (MDP) or similar infections of placebo (group 1 n=6). | Parasite detection in bone marrow aspirates. | Infection in dogs – presence of parasite after challenge of intravenous metacyclic promastigotes of *L.infantum.* |  | After challenge with injected *L.infantum* promastigotes: Groups 3 and 4 (100μg and 200μg of vaccine) were 100% (0/6 group 3 and 0/3 group 4) infection-free after both challenges. Group 2 (50μg of vaccine) saw 66.7% (2/6) infection from challenge 1, and 100% (6/6) of placebo dogs were found infected. |  | No P values reported. | Yes - 100μg of vaccine induced 100% protective efficacy. |
| Gradoni, 2005. Failure of a multi-subunit recombinant leishmanial vaccine (MML) to protect dogs from Leishmania infantum infection and to prevent disease progression in infected animals. | Phase III trial in dogs. Second generation vaccine. | 45 seronegative Beagle dogs (split into 3 groups of 15). | Italy. | Multi-subunit recombinant Leishmania polyprotein (MML, also known as Leish-111f). Each group received 3 monthly injections with vaccines A (MML+MPL-SE adjuvant), B (sterile saline=control) and C (MML+Adjuprime adjuvant). Surviving dogs received a second three-dose vaccine course 1 year later. | Every 2 months post vaccination, the following were obtained from each dog: (a) peripheral blood (PB) for spefic serology, immunology and clinical evaluation; (b) bone marrow (BM) aspirate for microscopy and leishmanial DNA detection by PCR (c) lymph node (LN) aspirate for parasite culture. | Cumulative incidence in dogs of infection (presence of parasites) after natural challenge with *Leishmania.* | Stage of infection. Comparative analysis of serological, parasitological and clinical findings allowed for characterisation of; subpatent infection, asymptomatic patent infection and symptomatic patent infection. | 1 year post-first vaccine course, cumulative incidence of leishmanial infections was 40% (6/15), 4 of which were subpatent and 2 asymptomatic patent infections) in group A, 43% (6/14) dogs showed subpatent infection only in group B and 36% (5/14), 4 subpatent and 1 asymptomatic patent infection in group C. No dogs from the three groups developed clinical signs of canine leishmaniasis. 2 year post-vaccination (1 year after the second vaccine course) the cumulative incidence was 87% (13/15) in group A (with three symptomatic cases), 100% (14/14) in group B (with no symptomatic cases) and 100% (10/10) in group C (with two symptomatic cases). | Among 15 infected animals, progression to a subsequent stage of infection was found in 5/6 dogs of group A, 3/6 of group B and 2/3 of group C. Cumulative incidence rates were not statistically significant between any groups at any time point. |  | No |
| Nogueira, 2005. Leishmune vaccine blocks the transmission of canine visceral leishmaniasis. Absence of Leishmania parasites in blood, skin and lymph nodes of vaccinated exposed dogs. | Randomised controlled trial. Dogs. Second generation vaccine. | 72 dogs – 40 untreated and 32 control. | Brazil. | Vaccination with Leishmune containing Fucose-Mannose ligand (FML) antigen of *L.donovani.* | Clinical symptoms and presence of parasites by PCR from blood and lymph node samples after natural challenge. | Clinical symptoms and presence of parasites in dogs by PCR from blood and lymph node samples. |  | PCR results from lymph node biopsies showed that 57% (17/30) control dogs were infected compared to 0% (0/18) vaccinated dogs were infected (P<0.005). VL symptoms showed that 25% (10/40) control dogs were symptomatic whereas 0% (0/32) from the treated group were symptomatic (P<0.005). |  | Not clear from the report as to why the denominator differs – results are not always out of the previously reported number of dogs (eg 40 in the untreated group, and 32 in the treated group). | No |
| Rafati, 2005. Protective vaccination against experimental canine visceral leishmaniasis using a combination of DNA and protein immunization with cysteine proteinases type I and II of *L.infantum.* | Randomised controlled trial. Dogs. Second generation vaccine. | 15 seronegative dogs (10 vaccinated with prime/boost with DNA/recombinant CPs, in combination with CpG ODN and Montanide 720) and 5 control dogs. | Iran. | 10 dogs received the prime/boost regimen of cysteine proteinases (CPs) type I and II. 1 dog did not receive and injection (environmental control), 2 dogs received saline injection, 2 dogs received the empty vector (no vaccine). | Bone marrow culture of parasites and PCR. | Numbers of dogs infected (parasites in bone marrow) after natural challenge. |  | 100% (10/10) vaccinated dogs did not show presence of parasite in bone marrow compared to 75% (3/4) control dogs which showed presence of parasites in bone marrow (P<0.001). |  | Natural challenge does not guarantee that all dogs will be exposed to infection. | Yes |
| Mohebali, 2004. Double-blind randomized efficacy field trial of alum precipitated autoclaved Leishmania major vaccine mixed with BCG against canine visceral leishmaniasis in Meshkin-Shahr district, I.R. Iran. | Randomised controlled trial. Dogs. First generation vaccine.** | 347 seronegative dogs. 182 received vaccine plus BCG, 165 received saline solution. | Iran. | Single dose of aluminium hydroxide (alum) precipitated autoclaved *L.major* (Alum-ALM) vaccine plus BCG against canine VL. | Seroconversion using DAT and ELISA and necropsy performed on dogs with clinical symptoms. | Infection (seroconversion) in dogs after natural challenge of 2 natural transmission seasons. |  | 3.7% (6/162) in vaccinated group and 12.0% (17/141) in control group using DAT technique. Similar results from ELISA. The efficacy of the vaccine was calculated to be 69.3%. |  | No P values reported. No other end points of VL infection reported. | Yes |
| Ramiro, 2003. Protection in dogs against visceral leishmaniasis caused by Leishmania infantum is achieved by immunization with a heterologous prime-boost regime using DNA and vaccinia recombinant vectors expressing LACK. | Randomised controlled trial. Dogs. Second generation vaccine. | 20 healthy (seronegative) dogs split into 4 groups. To test the prime-boost strategy of immunizing dogs with a plasmid carrying the gene for the LACK antigen from *L.infantum* followed by a booster of vaccinia virus. | Spain. | Group 1: negative control (saline), group 2: positive control (did not receive vaccination), group 3: vaccinated with two subcutaneous injections of the plasmid DNA-LACK (day 15 and day 30), group 4: two doses of the LACK encoding gene, one at day 30 with the plasmid DNA-LACK and a second, at day 15 with the vaccinia recombinant virus. | Tissue parasite load and development of clinical signs of leishmaniasis. | Presence of parasites in tissues and clinical symptoms in dogs. All dogs (except group 1 – negative control) were challenged on day 1 with *L.infantum* at day 0. |  | 0% (0/5) of group 1 had either parasites or clinical symptoms, 100% (5/5) of group 2 had both parasites and clinical symptoms, 80% (4/5) of group 3 gave clinical symptoms and 100% (5/5) dogs had parasites in tissues, 20% (1/5) dogs from group 4 presented clinical symptoms, and 40% (2/5) showed presence of parasites in tissue samples. |  | No P values reported. | No – reduction of parasite load conferred by vaccine is not enough to protect from infection. |
| Borja-Cabrera, 2002. Long lasting protection against canine kala-azar using the FML-QuilA saponin vaccine in an endemic area of Brazil (Sao Goncalo do Amarante, RN) | Phase III trial in dogs. Second generation vaccine. | 85 seronegative dogs included; 44 in the intervention group, 41 in the control group. | Brazil. | Intervention group received lyophilized FML antigen with QuilA saponin as adjuvant, control group received saline. | Presence of parasites was assayed in spleen, liver, kidneys, lymph nodes and bone marrow. | Infection rates in dogs within 41 months follow up (natural transmission challenge). | Death rates from VL. | After 41 months of follow up, 75% (3/4) of control dogs were positive for presence of parasite in smear tests compared to 0% (0/4) of the vaccinated dogs. No P value given. | 19.5% (8/41) of the control group of dogs and 2.3% (1/44) of the vaccinated group of dogs died as a result of VL infection during the study (P<0.05). |  | Yes |
| Molano, 2002. A Leishmania infantum multi-component antigenic protein mixed with live BCG confers protection to dogs experimentally infected with *L.infantum.* | Randomised controlled trial. Dogs. Second generation vaccine. | 20 seronegative Beagle dogs. 10 in intervention group (i.v Q protein+BCG), 10 in control group (i.v saline). | Spain. | Quimeric protein (Q), formed by the genetic fusion of five antigenic determinants from 4 Leishmania proteins, formulated with BCG. | LST and ELISA to test seroconversion. Parasite burden measured in lymph node aspirates and spleen post necropsy. | Infection in dogs after challenge with *L.infantum* promastigotes at 108 days post immunisation. 13 month follow up. |  | At day 150 -0st-infection parasites could be detected in 100% (10/10) of the control animals compared to 50% (5/10) of the vaccinated dogs. 3 of these became parasite negative at day 450 and 1 by the end of the study. On day 450, one of the control dogs became parasite negative and remained so until the end of the study. Final numbers of dogs with parasites are: 90% (9/10) of control dogs compared to 10% (1/10) in vaccinated dogs. | Data indicated that Q+BCG confers 90% protection against infection and at least 90% protection at the clinical level. | No P values reported. | Yes |
| Da Silva, 2001. A phase III trial of efficacy of the FML vaccine against canine kala-azar in an endemic area of Brazil (Sao Goncalo do Amarante, RN) | Phase III trial in dogs. Second generation vaccine. | 117 seronegative dogs were distributed in two groups . | Brazil. | Fucose mannose ligand (FML) vaccine of *L.donovani*: 58 received the FML-vaccine (3 injections) while 59 remained as placebo control treated with saline only (also 3 injections). | Clinical symptoms detected by veterinarians, and detection of parasite after necropsy. | Clinical signs in dogs or deaths due to kala-azar following natural transmission challenge. |  | 6.8% (4/59) dogs in the control group died of parasitologically confirmed VL during the first year of study, compared to 0% in the intervention group. 33% of the control animals developed either clinical or fatal disease while only 8% for vaccinated dogs showed mild signs of kala-azar with no deaths. The difference between the two groups is significant (P<0.0025). |  | Not all dogs were euthanized post study – only those with clinical symptoms. Actual data of numbers of dogs with clinical disease not reported. | Yes |
| Mohebali, 1998. Vaccine trial against into canine visceral leishmaniasis in the Islamic Republic of Iran. | Randomised controlled trial. Dogs. Second generation vaccine. | 16 dogs split into 4 groups of 4. | Iran. | Group 1 received autoclaved *L.infantum* with BCG, Group 2 received autoclaved *L.major* with BCG, Group 3, BCG alone and Group 4 received saline solution. Dogs received vaccines intradermally 3 times every 30 days. | LST, ELISA smear and culture after necropsy. | Seropositivity and presence of parasites after dog necropsy. Natural challenge. |  | 100% (4/4) dogs in both Groups 3 and 4 were seropositive by ELISA; and positive for presence of parasites after necropsy however none were positive for LST. 100% (4/4) of dogs in Group 1 were negative for ELISA and negative for presence of parasites after necropsy. 50% (2/4) were positive for LST. Group 2 showed 25% (1/4) positive for presence of parasites after necropsy, 75% (3/4) were positive for LST and 25% (1/4) was positive for ELISA. |  | Small sample numbers. No P values reported. | *L.infantum* plus BCG seems to be effective. |
| Mayrink, 1996. Phase I and II open clinical trials of a vaccine against Leishmania chagasi infections in dogs. | Randomised controlled trial. Dogs. First generation vaccine. | 19 dogs – 10 in intervention group (vaccine plus BCG), 9 9n unvaccinated control group. | Brazil. | Three doses of vaccine (600μg protein/dose) mixed with BCG (400μg/dose) were given intradermally at 21-day intervals to intervention group. | Culture, IFAT. | Seropositivity and presence of parasites after dog necropsy following intravenous challenge with promastigotes. |  | After necropsy, 10% (1/10) of unvaccinated dogs showed infection compared to 100% (9/9) of the control group. |  | No P values reported. | Yes |
| Dunan, 1989. Vaccination trial against canine visceral leishmaniasis. | Randomised controlled trial. Dogs. First generation vaccine. | 393 seronegative domestic dogs completed the study. | France. | Vaccine group received a lyophilized fraction of *L.infantum* with MDP adjuvant. Placebo group received sodium dodecyl sulphate and lactose with MDP. | Serology by IFAT. Seropositive dogs underwent lymph biopsy smear to check for presence of parasites. | Cases of VL in dogs over 2 years of follow up (natural challenge). |  | Cumulative incidence (over two year follow up) found 31 cases of seroconversion and 17 parasitologically confirmed cases in the intervention group, and 11 cases of seroconversion (of which 5 were confirmed through lymph smear) in the control group. No denominators are quoted for numbers. Authors conclude a statistically significant excess of cases of VL in dogs in the vaccine group (18.3%) compared to the control group (6.5%) P<0.001. |  | 39 dogs lost to follow up over 2 years. Authors do not specify which groups they came from. Numbers of dogs in each group are not revealed. | No. |

*Second generation vaccine defined as a recombinant protein or DNA vaccine and combinations of these.

**First generation vaccine defined as fractions of the parasite or whole killed Leishmania with or without adjuvants.
