# Supplementary material for: A Review of Preventative Methods against Human Leishmaniasis Infection
Source: PLoS Negl Trop Dis. 2013 Jun 20;7(6):e2278. doi: 10.1371/journal.pntd.0002278 (PMC3688540; doi:10.1371/journal.pntd.0002278)
Supplement: Dataset S2 — Full dataset of Vector population control studies. (DOCX) [file pntd.0002278.s002.docx]

Insecticide spraying studies

| Paper (author, title, year) | Study type | Number of subjects/areas | Location of study | Type of intervention | Method of diagnosis of infection | Primary outcome investigated | Secondary outcomes investigated | Main findings | Secondary findings | Comments | Intervention effective? |
| --- | --- | --- | --- | --- | --- | --- | --- | --- | --- | --- | --- |
| Chowdhury, 2011  Comparison of Insecticide-Treated Nets and Indoor Residuals Spraying to Control the Vector of Visceral Leishmaniasis in Mymensingh District Bangladesh | Cluster-randomized trial with four arms | 40 houses sampled monthly | Bangladesh | Indoor residual spraying (IRS) with deltamethrin compared to no intervention. | NA | Numbers of sandflies in household |  | 2 months after intervention 8 sandflies where collected in IRS houses compared to 54 in the control houses, rate ratio 0.15 (0.05-0.46) constituting a significant decrease (P<0.05). After 11 months 644 sandflies were collected per house compared to 451 in control houses RR 1.57 (1.09-2.25) constituting a significant increase (P< 0.05). |  | Good randomisation of houses | Yes, up to 11 months. |
| Coleman, 2011.  Impact of Phlebotomine Sand Flies on United States Military Operations at Tallil Air Base, Iraq: 6. Evaluation of insecticides for the Control of Sand Flies | Randomised Controlled Trial with 8 arms. | 8 areas randomly assigned to one of the 8 arms, each with untreated control areas. | Iraq | 1) routine sandfly control operations using a variety of residual and area-wide insecticides; 2) a combination of five different insecticide application methods in and around tents; 3) residual application of lambda-cyhalothrin and ultra-low volume application of pyrethrins in houses; 4) carbaryl and lambda-cyhalothrin applied as barrier sprays; 5) a deltamethrin impregnated fence; 6) lambda-cyhalothrin applied as a residual spray in concrete manholes; 7) deltamethrin-treated flooring in tents; and 8) ultra-low volume-applied malathion. Each of these arms were compared with an untreated control group. | NA | Sandfly Density |  | 1) routine sandfly control operations using a variety of residual and area-wide insecticides; no significant difference between the routine operations and control group (no P value given) 2) a combination of five different insecticide application methods in and around tents; F=3.35, P=0.0712. 3) residual application of lambda-cyhalothrin and ultra-low volume application of pyrethrins in houses; F=2.61, P=0.119, 4) carbaryl and lambda-cyhalothrin applied as barrier sprays; F=9.05, P=0.002, 5) a deltamethrin impregnated fence; F=0.76, P=0.395, 6) lambda-cyhalothrin applied as a residual spray in concrete manholes; F=0.25, P=0.629, 7) deltamethrin-treated flooring in tents; F=0.03, P=0.859, 8) ultra-low volume-applied malathion; no significant reduction in numbers of sandflies collected in light traps (no P value given). |  | Pretreatment surveillance was not conducted during one of the 8 studies. Although some of the experiments resulted in limited reductions in the number of sand flies collected in light traps, in no instance did they completely eliminate sand flies or reduce populations for a sustained period. | No. Arm 4 (carbaryl and lambda-cyhalothrin applied as barrier sprays) is the only intervention with a significant effect of decreasing sandfly numbers |
| Joshi, 2009. Chemical and environmental vector control as a contribution to the elimination of visceral leishmaniasis on the Indian subcontinent: cluster randomized controlled trials in Bangladesh, India and Nepal. | Cluster Randomised Controlled Trial with 4 arms | In each of four sites, four villages with high reported VL incidences were included. 16 clusters altogether. | 4 studies sites: Bangladesh (one), India (one) and Nepal (two) | Indoor residual spray (IRS): In Bangladesh deltamethrin was used, in India, DDT and in Nepal, alpha-cypermethrine | NA | Numbers of studies in household. |  | Results were pooled across countries. Control after 5 months mean sand flies per house per night = 12.15 (95% CI 8.68 – 17.00). IRS after 5 months = 6.14 (95% CI 4.00 – 10.47). IRS vs control p=0.035. |  | In each village six clusters and in each cluster five households were randomly selected for sand fly collection on two consecutive nights. Control and intervention clusters were matched with average pre-intervention vector densities. | Yes |
| Bray et al, 2009. Synthetic Sex Pheromone Attracts the Leishmaniasis Vector  Lutzomyia longipalpis (Diptera: Psychodidae) to Traps in the  Field. | Controlled trial. | Each experiment was conducted over two to four nights, using six to seven pairs of boxes  spread across two to three gardens. | Brazil. | Male sandfly synthetic hormone dispensed from mechanical light traps and sticky traps in gardens with or without the odor of chickens vs control traps using solvent only. | NA | Numbers of female and male sandflies caught in traps. |  | Using dispensers in conjunction with light traps and chicken odor, test traps with pheromone dispensers caught significantly more male and female sand flies than the solvent-only controls (P < 0.001). When sticky traps were used instead of light traps, more female (P <0.05) and male (P <0.01) sandflies were again captured at test stations than controls. |  | Approximately six  times more males than females were attracted to test and control stations. The authors conclude that his points to a skewed sex ratio in the trappable population at the field site, rather than a bias in response towards the synthetic pheromone. Numbers of sandflies caught are not reported. | Efficacy cannot be determined because numbers of sandflies caught is not reported |
| Das et al, 2009. Comparative study of kala-azar vector control measures in eastern Nepal | Random allocation, controlled intervention study with 4 arms | 1335 households. Total population covered was 6955 individuals. 24 clusters. After stratification of villages on the basis of disease incidence, four groups of six clusters were randomly allotted to the four arms of intervention; LLIN, IRS, EVM and Control. | Nepal | Alpha-cypermethrin Sprayed interior walls of the houses and cattle sheds up to six feet height covering all possible resting and breeding sites of sand flies. | NA | Sandfly densities. |  | IRS vs control : Vector density decreased from 11.0 to 0.6 per house per night (p=0.009) |  | Large differences in baseline between intervention and control arms which could affect results. | Yes |
| Souza et al, 2008 Communitary assay for assessment of effectiveness of strategies for prevention and control of human visceral leishmaniasis in the Municipality of Feira de Santana, State of Bahia, Brazil (Portuguese language paper). | Controlled trial with three arms | Three villages containing 688, 782 and 892 infants (9 mo 12 yrs) respectively each into each of the three interventions. | Brazil | Group A: control, group B; spraying houses with deltamethrin, group C; spraying houses with deltamethrin and elimination of infected dogs to control | Montenegro Skin Test (MST) to check initial reactivity and ten ELISA survey every 12 months. | Incidence of infection of VL in children < 12 years |  | During the total 2 years follow up, Group A saw 2.74% (13/475) incidence, Group B 2.51% (14/558) incidence, Group C 1.94% (12/628). Relative risk for infection the areas with spraying only vs control was 0.99 (95% CI: o.46-2.10), spraying ad dog culling vs control was 0.74 (95% CI 0.34-1.62); neither of these difference was statistically significant (no P values given) |  | No P values reported | No |
| Nery Costa et al, 2007. Controle da leishmaniose visceral em meio urbano: estudo de intervenção randomizado factorial (Portuguese language paper). | Controlled trial | 213 seronegative individuals included in study. The area was divided in 34 blocks alternately allocated to four types of intervention. | Brazil | 1) spraying houses and animal pens with insecticide; 2) spraying houses and eliminating infected dogs; 3) combination of spraying houses and animal pens, and eliminating infected dogs, and 4) control group (only spraying houses). | Humans seroprevalence (by ELISA). Dog seroprevalence (by indirect immunofluorescence (IFAT)) used to detect seropositive dogs to be eliminated. | Human VL prevalence. |  | Spraying houses and animal pens was associated with a (non-significant) decreased incidence of visceral leishmania compared to “no intervention” control (which actually consisted of just house spraying) OR 0.64 9% CI 0.19-2.18). No P values reported. Of the 213 seronegatives included, only 56% (120) were analysed after 6 months to test seroconversion. Of these 38.7% (46/120) became infected during 6 months after being in the spraying only intervention group. |  | Seems to have a large dropout – Only 56% of seronegatives were followed up study with. Study to be under-powered. The 213 seronegatives, of which 120 were re-tested are not described by intervention group therefore it is impossible to tell the dominator of how many seropositives there were in each group. We only have percentages. | No |
| Feliciangeli et al, 2003. Control trial of Lutzomyia longipalpis s.l. in the Island of Margarita, Venezuela. | Controlled trial | 1 control village and 1 intervention 5 houses from each village | Margarita Venezuela | Intradomestic residual spraying (IRS) of lambda-cyhalothrin, every 5 months and spatial fogging of fenitrothion around the houses. 16 foggings during the year. | NA | Sandfly abundance |  | Treatment vs control indoor sandfly density (females): 146/360 (P=0.000) outdoor (females): 1168/2083 (P=0.000) | Spatial fogging not recommended as method is extremely weather-effected. Only recommended in epidemic situations. | The increase in sandflies density after treatment could be caused by movement of sandflies to the other area? Spatial fogging expensive and as transmission occurs indoors, fogging might do more harm than good with respect to emerging resistance. | Yes |
| Feliciangeli et al, 2003. Cutaneous leishmaniasis vector control perspectives using lambdacyhalothrin residual house spraying in El Ingenio, Miranda State, Venezuela. | Randomized Controlled Trial | Houses – control= 19, treated = 20 | Venezuela | Indoor Residual Spraying (IRS) of houses with lambdacyhalothrin | NA | Sandfly abundance | Fed females | Significant reduction in bloodfed female sandflies caught in sprayed houses immediately following insecticide application (5.8% in control houses compared to 0-8% in sprayed houses P<0.001). Total mean catch of females and males in control compared to treatment houses after intervention: females control: 6.4, females treatment 1.9 (P<0.0005). Males control 1.5, males treatment 0.1 (P<0.0005). Numbers of sandflies recovered, reaching control levels by 7 weeks after intervention. | Short residual effect of insecticide. |  | Yes |
| Reyburn et al, 2000. A randomized controlled trial of insecticide-treated bednets and chaddars or top sheets, and residual spraying of interior rooms for the prevention of cutaneous leishmaniasis in Kabul, Afghanistan. | Cluster Randomized Controlled Trial with 4 arms | Non-immune study population of 3666 people. | Kabul Afghanistan | Household residual pyrethroid (lambdacyhalothrin) spraying compared to no interventions | Questionnaire completed by parents of the  household, visual checking of reported cases by person familiar with the disease. No culture or smear was taken | Self-reported cases of ACL | Adverse reactions, popularity of interventions | At the end of the trial period (15 months) the incidence of ACL amongst controls was 7.2% (92/1281) amongst residents of sprayed houses 4.4% (36/813) (OR 0.60, 95% CI 0.3-0.95) P=0.031 | No significant differences for age or sex were found between new cases in the intervention and control groups. No serious side-effects reported. Interventions were generally popular; ITNs were the most popular followed by residual spraying and then impregnated chaddars. | IRS works better when all houses sprayed. Possible effect of diverting sandflies onto unprotected people sleeping nearby was not investigated. Data on use of nets was self-reported and so compliance uncertain . About 45% of study population moved during study but this was anticipated due to violence and sample size was made adequately large with this in mind. | Yes |
| Davies et al, 2000. Spraying houses in the Peruvian Andes with lambda-cyhalothrin protects residents against cutaneous leishmaniasis | Randomized Controlled Trial | 112 intervention houses and 154 control houses (closely matched pre-intervention measurements of incidence & sandfly abundance) 437 total seronegative individuals. | Peruvian Andes | Spraying walls and ceilings with lambda-cyhalothrin 1992-1993. | Leishmania Skin Test (LST) and clinical symptoms | Sandfly abundance | Cases of CL | Comparisons of pre- and post-intervention sandfly indoor abundance, measured at regular intervals for up to 2 years using light traps, in 22 sprayed & 21 control houses demonstrated that spraying significantly reduced the indoor abundance of Lu. verrucarum by an average of 78.2% (34.8-92.7) P=0.006 & of Lu.(Helcocyrtomyia) peruensis by 83.5% (28.9-96.1) P=0.016. Spraying did not significantly reduce the density of L. noguchii (P=0.08) | Prevalence of 10% (24/21) of cases of CL in the control houses compared to 4.6% (9/196) in the intervention houses. This equate to a reduction of 54% (95% CI 4.0-76.6% P=0.039) | Well controlled, pre-intervention risk was well-matched. When incident CL cases diagnosed in the first 3 months were excluded from analysis, the effect was even greater (P=0.020) - the authors assume that cases within the first three months might have been caused by an infection prior to the start of the intervention | Yes |
| Kelly et al, 1997. Differential application of lambda-cyhalothrin to control the sandfly Lutzomyia longipalpis. | Randomised Controlled Trial | 30 villages - 10 houses with chicken sheds in each. Chicken sheds were randomly assigned to one of three treatments: 1. spray, 2. sprayed sheets hung in sheds or 3. control (no insecticide). | Brazil | Lambda-cyhalothrin applied as a spray to chicken sheds or sheets attached inside chicken sheds compared to untreated controls | NA | Sandfly densities |  | The fall in abundance of sandflies in sprayed sheds was significantly greater than in sheds with sprayed sheets (P < 0.025; n = 20). Abundance of other species of sandfly did not decrease significantly (P>0.05) in sprayed or huts with sheets. Analysis of numbers of sandflies in huts used by humans for dining showed no significant decrease in sandfly numbers in either intervention group compared to control sheds (p>0.05). |  | Author suggests that spraying of external building diverted sandflies away from those and into buildings used by humans for dining. | No |
| Alexander et al, 1995. An attempt to control phlebotomine sand flies (Diptera, psychodidae) by residual spraying with deltamethrin in a Colombian village. | Matched Paired controlled trial | Twelve houses were divided into matched pairs based on physical characteristics, one house in each pair being left untreated while the other was treated | Cali, Colombia | Deltamethrin sprayed on the inside walls of houses | NA | Sandfly densities |  | The number of sand flies (Lutzomyia youngi) collected on sticky traps was significantly lower in the untreated control houses (63) than in the treated ones (546) with which they were matched P=0.004. The number of L youngi collected on human bait in treated vs untreated houses was not significantly different. |  | No decrease in sandflies associated with treatment - This may have been the result of sand flies entering and biting before making contact with the treated surface. Confounding factors are suggested such as the insecticide causing sandflies to fly erratically and more likely to be caught. | No |
| Kaul et al, 1994. Impact of DDT indoor residual spraying on Phlebotomus argentipes in a kala-azar endemic village in eastern Uttar Pradesh | Controlled Trial | 2 villages – one sprayed with DDT, one control unsprayed | Uttar Pradesh, India | DDT indoor spraying compared to unsprayed control village | NA | Numbers of female male sandflies captured in houses |  | Female sandflies as a proportion of total captured; 50% (7/14) (females/total) in treatment area vs 79% (289/365) (females/total) in control area. No P values given. Authors conclude that DDT spraying is effective in decreasing vector numbers |  | No statistical tests performed | Yes but no significance tests reported |
| Benzerroug et al., 1992. Les pulverisations intra-et péri-domociliaires de DDT dans la lutte contre la leishmaniose cutanée zoonotique en Algérie. (French language paper). | Controlled Trial | 2 villages –one sprayed with DDT, one control untreated. Spraying carried out in May 1983. | Algeria | DDT indoor and outdoor spraying compared to unsprayed control village. | NA | Number of sandflies captured in houses |  | During 4 captures in 5 months following DDT treatment, a total of 377 sandflies were captured in the control village traps compared to 82 in the DDT sprayed village. |  | No statistical tests performed. Baseline sandfly densities not taken into account but the authors note a higher baseline in the treated village prior to intervention. | Yes but no significance tests reported. |
| Jacusiel et al, 1947. Sandfly control with DDT Residual Spray. Field experiments in Palestine. | Non-randomised matched controlled trial. | 5 houses - one room sprayed with DDT, other rooms left untreated. | Palestine. | Spraying walls and ceiling with DDT. | NA – none available in 1947 | Number of sandflies in room. | Incidence of “sandfly fever”. | Authors conclude that DDT spraying is effective in reducing the number of sandflies. The residual action lasted for 52-58 days. The sandfly-fever rate in 1945 (after spraying) was much lower (approximately one-tenth) than in 1944 (no spraying). No P values given. | Secondary DDT effect of adjoining rooms, Protection afforded by DDT residual spray was equally satisfactory against all 3 species, *P. papatasi*, P. major and *P. chinensi*. | DDT shown to be effective – no side effects of DDT investigated. | Yes but no significance tests or P values given. |

Environmental management studies

| Paper (author, title, year) | Study type | Number of subjects/areas | Location of study | Type of intervention | Method of diagnosis of infection | Primary outcome investigated | Secondary outcomes investigated | Main findings | Secondary findings | Comments | Intervention effective? |
| --- | --- | --- | --- | --- | --- | --- | --- | --- | --- | --- | --- |
| Chowdhury, 2011 Comparison of Insecticide-Treated Nets and Indoor Residual Spraying to Control the Vector of Visceral Leishmaniasis in Mymensingh District, Bangladesh. | Cluster-randomised trial with four arms. | 40 houses sampled monthly. | Bangladesh. | Mud plastering of wall and floor cracks, | NA not an outcome. | Numbers of sandflies in household. |  | The mud plastering of wall and floor cracks showed no impact on sandfly numbers. 2 months after intervention, 43 sandflies were collected in the intervention arm houses compared to 54 in the control arm. Rate ratio 0.88 (95% CI 0.45-1.69). There was no significant difference between the intervention and control arms until 11 months post-intervention when sandfly densities surpassed control; 598 sandflies collected in the intervention arm compared to 451 in the control arm (RR 1.43 (95% CI 1.00-2.06 P<0.05). |  | Good randomisation of houses. | No |
| Das, 2009. Comparative study of kala-azar vector control measures in eastern Nepal. | Randomised controlled trial. | 1335 households. Total population 6955. 24 clusters. | Nepal. | Plastering the inner walls of houses with lime. | NA not an outcome. | Household sandfly densities. |  | Lime plastering vs control: Vector density decreased from 8.2 to 2.6 per house per night (p=0.785). |  | Large differences in baseline between intervention and control arms which could affect results. | No |
| Joshi, 2009. Chemical and environmental vector control as a contribution to the elimination of visceral leishmaniasis on the Indian subcontinent: cluster randomized controlled trials in Bangladesh, India and Nepal. | Cluster randomized controlled trial. | 96 clusters (hamlets or neighbourhoods). | 4 study sites: Bangladesh (one), India (one) and Nepal (two), In each site four villages with high reported  VL incidences were included. | Environmental management arm consisted of mud plastering in Bangladesh and lime plastering in Nepal and India. | NA not an outcome. | Numbers of sandflies in household. |  | Results were pooled across countries. Control after 5 months mean sandflies per house per night = 12.15 (95% CI 8.68 - 17.00). EVM after 5 months = 10.39 (95% CI 7.56 - 14.29). Intervention vs control p=0.503. Mud plastering did not reduce sand fly density (Bangladesh study); lime plastering in India and one Nepali site, resulted in a significant reduction of sand fly density but not  in the second Nepali site. |  | In each village six clusters and in each cluster five households were randomly selected for sand fly collection on two consecutive nights. Control and intervention clusters were matched with average pre-intervention vector densities. Data pooled over countries. | Pooled results conclude that the intervention is ineffective but at a county level, 2/4 sites showed a significant decrease in sandfly numbers and 2/4 sites did not. |
| De Magalhaes, 2009. Dissemination of information on visceral leishmaniasis from schoolchildren to their families: a sustainable model for controlling disease. * | Randomised controlled trial. | 2 schools (1 intervention n=92, 1 control n=96 students). | Belo Horizonte, Miras Gerais State, Brazil. | Education – intervention= visceral leishmaniasis class given by teacher, a copy of the pamphlet + students received instructions on how to spread the information to their families. Control= visceral leishmaniasis class given by the teacher, a copy of the pamphlet. | NA not an outcome. | Retention of knowledge 90 days after class compared to before the class. Family knowledge evaluated after 30, 90 and 120 days. | Evaluated hygiene around the household (leaves, fruit, or branches on the ground, accumulated garbage, and animal droppings). | Similar low knowledge of students prior to class. After class, average percentage of correct answers in the intervention group was 53.3%, as compared to 39.2% in the control group. Significant difference in improvement (P<0.05). Family members intervention 51.1% to 71% 120 days after. Control 43.5% to 52.2% (P<0.05). there were no differences in peridomiciliary hygiene before the intervention. At 30 and 90 days, the intervention group showed significant improvements in sweeping of leaves, fruits and branches (P<0.05). |  |  | Yes |
| Kumar et al, 1995. Field trial of an ecological approach for the control of  Phlebotomus argentipes using mud and lime plaster. | Controlled intervention study. | 10 houses, including the cattle sheds and latrines, were treated and 5 were untreated controls. | India. | Plastering of houses (including cattle sheds and latrines) with mud and lime up to a height of 1.22 m. | NA not an outcome. | Sandfly density in houses. |  | Decrease in sandfly density in treated houses. No significant drop in density in untreated houses. In 7 months follow up post treatment, 104 sandflies per man hour were collected in the control houses compared to 13.76 in the control houses. The difference between the mean values is significant (p<0.05). |  |  | Yes |
| Robert and Perich, 1995. Phlebotomine sand fly (Diptera: Psychodidae) control using a residual pyrethroid insecticide. | Controlled study. | 2 settlements - one control, one intervention site. | Baringo district, Kenya. | Termite mounds and animal burrows were treated with a cyfluthrin/corn oil mixture as insecticide. | NA not an outcome. | Numbers of sandflies in settlement. |  | Cyfluthrin provided significant residual control of sandflies for 12 weeks in termite mounds (P=0.01) and animal burrows (P=0.01) compared to the control site. No data given. |  | No data reported. | Yes |

* de Magalhaes, 2009 study is included in the Environmental Management group as the intervention (education) pertains to knowledge of environmental risk factors such as keeping household clear of organic debris.
