# Supplementary material for: A Review of Preventative Methods against Human Leishmaniasis Infection
Source: PLoS Negl Trop Dis. 2013 Jun 20;7(6):e2278. doi: 10.1371/journal.pntd.0002278 (PMC3688540; doi:10.1371/journal.pntd.0002278)
Supplement: Dataset S3 — Full dataset of Human reservoir control studies. (DOCX) [file pntd.0002278.s003.docx]

Untreated and insecticide-impregnated nets and fabrics

| Paper (author, title, year) | Study type | Number of subjects/areas | Location of study | Type of intervention | Method of diagnosis of infection | Primary outcome investigated | Secondary outcomes investigated | Main findings | Secondary findings | Comments | Intervention effective? |
| --- | --- | --- | --- | --- | --- | --- | --- | --- | --- | --- | --- |
| Chowdhury et al, 2011. Comparison of Insecticide-Treated Nets and Indoor Residual Spraying to Control the Vector of Visceral Leishmaniasis in Mymensingh District, Bangladesh. | Cluster randomised trial with four arms. | 296 households. | Bangladesh. | Deltamethrin insecticide – treated bed nets* (ITNs) compared to no intervention. | NA not studied. | Numbers of sandflies in household. |  | Until the end of the study (11 months after intervention), the rate ratio of all sandflies in households in ITN compared to control houses was decreased. After 2 months: 0.36 (0.15-0.83) after 4 months; 0.26 (0.18-0.37) after 5 months 0.22 (0.14-0.33), and 0.40 (0.26-0.60) after 11 months. These reductions in sandfly density are all significant at P<0.05. Vector density did not rebound back to control levels. |  | Details of house randomisation given. Permanet 2.0 nets used have minimum 156 holes/in^2^ | Yes |
| Gidwani et al, 2011. Serological markers of sand fly exposure to evaluate insecticidal nets against visceral leishmaniasis in India and Nepal: a cluster-randomized trial. | Paired cluster-randomised trial. | 305 people from 26 VL-endemic clusters. | India and Nepal. | Deltamethrin-impregnated bed nets compared to an unspecified ‘control’ group. | NA not studied. | Effect of ITNs on sand fly biting by measuring the antibody response of subjects to the saliva of vector *P. argentipes* or non-vector *P. papatasi*. |  | Random effect linear regression model showed that cluster-wide distribution of ITNs reduced exposure to *P. argentipes* by 12% at 12 months (0.88 (95% CI 0.83-0.94) P<0.001) and 9% at 24 months (0.91 (95% CI 0.80-1.02) P=0.115) in the intervention group compared to control adjusting for baseline values and pair. Similar results obtained for *P.papatasi*: 12 mo; 0.89 (95% CI 0.82-0.96) P=0.002, 24 mo; 0.91 (95% CI 0.84-0.99) P=0.034. | Supports the use of sandfly saliva antibodies as a marker to evaluate vector control interventions. | Authors conclude that ITNs did not confer significant additional protection against sandfly bites in VL-endemic regions and question the indoor transmission of *L.donovani* in these regions. The study does not specify what the control group was (no intervention or untreated nets). Bed nets used have minimum 156 holes/in^2^ | Yes |
| Faiman et al, 2011. Exclusion of phlebotomine sand flies from inhabited areas by means of vertical mesh barriers. | Controlled trial. | 6 houses - three near the barrier and three far from the barrier. | Jordan Valley, Israel. | A 400 meter long section of the boundary of a settlement was draped with a deltamethrin-impregnated net that is impenetrable to sand ﬂies. | NA not studied. | Sandfly numbers inside/outside barrier. |  | In the barrier group an 84.9% decrease was noted (19.93 sand ﬂies/trap/night,  before and 2.60 sand ﬂies/trap/night after erection of the barrier (P = 0.003). Concurrently, the neighbouring control group of three houses, not protected by the barrier, exhibited a 15.9% increase in sand ﬂy numbers (6.99 before and 7.02 sandﬂies/trap/night after erection of the barrier (P = 0.974)). |  | Barrier used had 450 holes/in^2^ | Yes |
| Mondal et al, 2010. Insecticide-treated bed nets in rural Bangladesh: their potential  role in the visceral leishmaniasis elimination programme. | Non-randomised controlled trial. | 50 intervention households and 26 control households. | Bangladesh. | Deltamethrin-impregnated bed net study. | NA | Sandfly densities at baseline, 1, 12 and 18 months. Measured with light traps. |  | Mean difference from baseline between the intervention and control areas at 1 month was not statistically significant. At 12 and 18 months the mean difference from baseline between intervention and control areas was significant, P=0.047 and P=0.024 respectively. At 12 months intervention area mean difference from baseline = -8.9 95%CI -14.48 to -3.32, control area = 01.08 95%CI -02.43–04.58. At 18 months, intervention area mean difference from baseline = -11.28 95% CI -16.71 to -5.85, control area = -0.27 95% CI -3.63–3.10. |  | Power calculations were conducted to determine numbers of houses needed for follow up. Baseline sandfly densities were different between intervention areas were different (intervention mean per house per night; 17.68 95%CI 16.53–18.88, and control mean per house per night; 09.88 95%CI 08.71–11.79) however P values are given on the mean difference from baseline. | Yes |
| Picado et al, 2010. Longlasting insecticidal nets for prevention of Leishmania donovani infection in India and Nepal: Paired cluster randomised trial. | Paired cluster randomised. | 13 intervention and 13 control clusters - 12,691 individuals (main outcome - infection) and 19,810 (secondary outcome - disease). | India and Nepal. | Deltamethrin-treated insecticide nets compared to any existing personal protection used by individuals. | DAT at 12 and 24 months. | Human seroconversion. | Human clinical disease. | No significant difference in the risk of seroconversion over 24 months in intervention clusters (5.4% 347/6372) compared to control (5.5% 345/6319) RR 0.90 (95% CI 0.49-1.65). P= 0.71. | No significant difference in the risk of clinical visceral leishmaniasis between intervention 0.38% (37/9829) and control 0.40% (40/9981) clusters (RR 0.99 95% CI 0.46-2.16). P=0.99. | Permanet 2.0 nets used which have a minimum 156 holes/inch^2^ | No |
| Picado et al, 2010. Effect of Village-wide Use of Long-Lasting Insecticidal Nets on Visceral Leishmaniasis Vectors in India and Nepal: A Cluster Randomized Trial. | Cluster randomised controlled trial. | 3 intervention and 3 control clusters with 10 houses per cluster (120 houses in total). | India and Nepal. | Insecticide-impregnated bed nets compared to normal practise of protection. | NA | Sandfly densitites (specifically *P.argentipes).* |  | A random effect linear regression model showed that the cluster-wide distribution of ITNs significantly reduced the *P. argentipes* density/house by 24.9% (95% CI 1.80%–42.5% P=0.036) as measured by means of light traps. |  | 2 out of 120 households initially included in the trial were lost to follow up. Between 46 and 64% of houses used untreated nets before the trial. Since no attempt to stop this was made due to ethical reasons, the control group may have been using other forms of personal protection. Insecticide used for the bed nets is not specified. Permanet 2.0 nets used have a minimum 156 holes/inch2. Tables and figures are presented as such that numbers could not be extracted for analysis. | Yes |
| Das et al, 2009. Comparative study of kala-azar vector control measures in eastern Nepal. | Cluster randomised controlled trial. | 1335 households included. The total population covered was 6955 in 24 clusters. | Nepal. | After stratification of villages on the basis of disease incidence, four groups of six clusters were randomly allotted to the four arms of intervention; deltamethrin-impregnated bed nets, IRS, EVM and Control (no intervention). | NA | Sandfly densities. |  | ITN vs control: Vector density dropped from 2.3 (95% CI 1.1-4.9) in the control to 0.9 (95% CI 0.6-1.3) in intervention houses per house per night (p=0.019). |  | Large differences in baseline between intervention and control arms which could affect results. Permanet 2.0 nets used have a minimum 156 holes/inch^2^. | Yes |
| Joshi et al, 2009. Chemical and environmental vector control as a contribution to the elimination of visceral leishmaniasis on the Indian subcontinent: cluster randomized controlled trials in Bangladesh, India and Nepal. | Cluster randomised controlled trial with 4 arms. | Four sites, four villages in each site with high reported  VL incidences were included. | Four study sites: Bangladesh (one), India (one) and Nepal (two). | Deltamethrin-impregnated bed nets compared to no intervention. | NA | Numbers of sandflies in household. |  | Results were pooled across countries. Control after 5 months mean sandflies per house/night was 12.15 (95% CI 8.68 - 17.00). LLIN was 8.32 (95% CI 5.56 - 12.45). LLIN vs control P=0.160. Authors state that LLINs had a significant effect in India and Bangladesh but not in the two Nepali sites. |  | The light traps used to collect sandflies were located outside the bed nets and so may not give an accurate indication of protection against bites beneath the net. Bed nets used have minimum 156 holes/inch^2^. Individual country data compared to control is not reported. | Overall no, however authors state a significant reduction in sandfly numbers in 2/4 sites, and a significant increase in 2/4 sites. No raw data reported. |
| Picado et al, 2009. Effect of untreated bed nets on blood-fed Phlebotomus argentipes in kala-azar endemic foci in Nepal and India. | Non randomised controlled trial. | 58 households in six clusters (10 households per cluster) for intervention. Six clusters of 10 households for control. | India (6 clusters) and Nepal (6 clusters). | Untreated nets compared to no intervention. | NA | Blood feeding rates of *P. argentipes* VL endemic villages. |  | The use of untreated nets reduced the blood feeding rate by 85% (95% CI 76.5-91.1% P<0.001) from 21.5% (114/530 – blood fed/total females) to 2.7% (29/1074) for all intervention groups combined. |  | Net hole size not reported. All houses in the study were given insecticide-treated nets but on the nights of collection for this study, certain houses were given untreated nets. Treated nets were returned to households the following morning. | Yes |
| Faiman et al, 2009. Control of Phlembotomine Sand Flies with vertical Fine Mesh Nets. | Non-randomised controlled trial | NA | Jordan Valley, Israel | Deltamethrin-impregnated barrier net (225 holes/inch^2^) compared to beta-cyfluthrin sprayed net (1,240 holes/inch^2^) compared to adjacent unprotected areas | NA | Sandfly penetration of nets |  | Both net types exhibited >90% efficacy in blocking sand flies from entering the enclosures (P < 0.01). The 1,240 holes/inch^2^ net exhibited high efficiency even before being sprayed with insecticide because the small mesh size physically prevented flies from passing through. Sand flies were monitored on all sides of the barrier using CO2-baited CDC traps or CDC light traps. Results showed a 60% reduction in the mean number of sand flies trapped behind the net compared with the untreated areas adjacent to it (P < 0.05). |  | Barrier nets were 60m-long and 2m-high | Yes |
| Dinesh et al, 2008 Long-lasting insecticidal nets fail at household level to reduce abundance of sandfly vector Phlebotomus argentipes in treated houses in Bihar (India). | Controlled trial. | 3 hamlets, 16 houses in each Hamlet. Half untreated nets, half treated nets. Equal number of mixed (cattle and humans) and human only dwellings. | Bihar, India. | Longlasting deltamethrin-treated insecticide nets (Olyset and PermaNet vs untreated net). | NA | Sandfly population inside houses. |  | Neither ITN demonstrated any impact at household level of female *P. argentipes* densities compared to untreated net (Incidence ratio permanet vs control 0.68 P=0.40, Olyset vs control 0.79 P=0.61) No difference in mixed or human-only dwellings. |  | No raw data given. No mention of randomisation. Baseline sandfly numbers were taken into consideration in analysis. Olyset nets have 50 holes/inch^2^. Permanet 2.0 nets have a minimum of 156 holes/inch^2^. | No |
| Jalouk et al, 2007. Insecticide-treated bednets to prevent anthroponotic cutaneous leishmaniasis in Aleppo Governorate, Syria: results from two trials. | 1997—1999 matched-cluster randomised trial (5  intervention (ITNs) and 5 control (untreated bednets) villages). The second study (2001—2003) was not controlled and nets could not be replaced or re-impregnated due to limited resources. The 2001—2003 study is not discussed here. | 10 354 people in 1321 households. | Aleppo, Syria. | Deltamethrin-impregnated bednets vs untreated bednets (control). | Active case finding conﬁrmed parasitologically through dermal slide smears. | ACL incidence. |  | Incidence of new lesions in treated villages decreased from baseline of 1.7% to 0.5% (19/4010). In control villages, rate of new lesions increased from baseline of 2.6% to 3.4% (116/3410). Protective efficacy estimated from pooled results of treated compared to control villages was 85% (95%CI 76-98) P=0.05. |  | Variation between control groups. Some saw increased incidence, some a reduction (overall there was an increased incidence). Different intensities of ACL transmission may have occurred in the two regions during the 1998 season. Tables and figures are presented as such that numbers could not be extracted for analysis. 156 holes/inch^2^ bed nets were used. | No |
| Courtenay et al, 2007. Deltamethrin-impregnated bednets reduce human landing rates of sandfly vector Lutzomyia longipalpis in Amazon households. | Crossover study. | 4 houses. 2 treated bednets, 2 untreated bednets in each. | Marajó Island, Pará State, Brazil. | Deltamethrin impregnated bednets compared to untreated bed nets. | NA | Human landing rates of *Lutzomyia longipalpis*, 24-h mortality rate. | Landing rates and mortality (protection conferred) of unprotected houses/rooms). | Mean sandflies collected per night under treated nets was 1.3 (95%CI 0.84-2.05) which is significantly fewer than the mean collected under untreated nets of 2.6 (95%CI 1.78-3.85) P<0.023. Reduction in sandfly landing rates from 71% (95%CI 60-81) in untreated nets to 14.5% (95%CI 6-31) in treated households. P<0.0001. 24-h mortality increased to to 68% (95% CI 62-73%) in treated houses compared to 0.4% (0-2%) in untreated houses P=0.001. | The presence of an ITN also reduced the human landing rate on unprotected persons outside the net in the same room by 56% (95% CI  52-59%) from 49% (95%CI 44-53) in untreated rooms to 22% (95%CI 18-265) P<0.001. | SmartNet has 400 holes/inch^2^ - just large enough to allow sandflies through. | Yes |
| Motavalli -Emami, 2006. Impact of Olyset long lasting nets on anthroponotic cutaneous leishmaniasis in Islamic Republic of Iran. | Randomised cluster trial. | A total of 8620 individuals in 3000 households. Twelve urban sectors of two cities with ACL  were selected. Six sectors were randomly  selected into intervention and control areas. | Iran. | Permethrin-treated bed nets vs no intervention. | Surveys to detect new cases of ACL. | Cases reported by survey of ACL. |  | There was a significant reduction in ACL incidence in both areas; intervention area compared to the control site 1: (0% compared to 0.88%) P<0.05, intervention area compared to control site 2: 0.09% compared to 5.1% P<0.05. Relative risk 0.02 (95%CI 0.00-0.07) P<0.05. |  | Olyset nets with wider mesh size (50 holes/inch^2^) may be preferred by the community in endemic areas due to increased ventilation. Raw data not reported. | Yes |
| Alten et al, 2003. Evaluation of protective efficacy of K-OTAB impregnated bednets for cutaneous leishmaniasis control in Southeast Anatolia-Turkey. | Controlled trial. | 10,468 people - in 5 areas. | Anatolia, Turkey. | Deltamethrin-impregnated bednets (2 areas), untreated bednets (1 area) or nothing (2 areas). | Not stated. | Incidence of ACL. |  | Significant (P<0.05) reduction in cutaneous leishmaniasis incidence in the intervention areas from 1.87% (44/2242) to 0.035% and from 2.3% (33/1519) to 1.32%. incidence increased in one of the control areas from 1.03% (16/1554) to 1.38%, the other there was no change. The area with the untreated bednets increased from 1.22% (14/1071) to 1.47%. Only raw data were given for pre-intervention data. Post-intervention data was only reported as percentages. |  | No mention of how a case was confirmed. Nets used had 1000 holes/inch^2^. No pooled estimates of effect for combined intervention vs combined control were presented. | Yes |
| Asilian et al, 2003 Efficacy of permethrin-impregnated uniforms in the prevention of cutaneous leishmaniasis in Iranian soldiers. | Randomised controlled trial. | 324 soldiers randomised into two equal groups - 272 completed the study. 134 in group A wore permethrin-impregnated uniforms. group B, 138 soldiers were issued uniforms washed in water. | Isfahan, Iran. | Human insecticide-impregnated uniforms worn for 3 months. Soldiers observed for 6 months after. All the soldiers remained in the leishmania-endemic area of Isfahan during the 3 months. | Diagnosis  of CL was conﬁrmed in every suspected lesion  parasitologically using Giemsa-stained direct  smears, and if amastigotes were not seen, the lesion  was biopsied. | Cases of Cutaneous Leishmaniasis |  | 6.5% (9/138) soldiers wearing control uniforms and 4.4% (6/134) soldiers wearing permethrin-impregnated uniforms acquired CL. The difference between two groups was not statistically significant (P < 0.05). |  | Double blind trial design. Permethrin-impregnated uniforms are not effective for the prevention of CL. | No |
| Kroeger et al, 2002. Insecticide impregnated curtains to control domestic transmission of cutaneous leishmaniasis in Venezuela: cluster randomised trial. | Cluster randomised controlled trial. | 2913 inhabitants of 569 houses. | Trujillo, Venezuela. | Intervention group received curtains impregnated  with lambdacyhalothrin while the control groups received curtains without insecticide  or no curtains. After 12 months a follow up household survey was conducted. | Questionnaire completed before and 12 months after. Cases detected in a clinic. Not stated how leishmania was detected. | Self-reported 12 month incidence of clinical  cases of CL. | Reduction in abundance of sandflies indoors. | 12 months after intervention, incidence of cutaneous leishmaniasis  was 0% (0/1294) in the intervention group and 8% (85/1103) in the six pairs in the control group that received non-impregnated curtains (mean difference 8, 95% CI 4.22 to 11.78; P=0.001). | There were significantly fewer sandflies captured in the intervention group (mean of 2 per trap) compared to the control group (mean of 15 per trap) P<0.001. |  | Yes |
| Reyburn et al. 2000. A randomized controlled trial of insecticide-treated bednets and chaddars or top sheets, and residual spraying of interior rooms for the prevention of cutaneous leishmaniasis in Kabul, Afghanistan. | Randomised controlled trial with 4 arms. | Non-immune study population of 3666 people. | Kabul, Afghanistan. | Compare the efficacy of permethrin-treated bed nets (ITNs), permethrin-treated bed sheets (chaddars), and no intervention. | Questionnaire completed by parents of the  household, visual checking of reported cases by person familiar with the disease. No culture or smear was taken. | Self-reported clinical cases of ACL. | Adverse reactions, popularity of interventions. | At the end of the trial period (15 months) the incidence of ACL amongst controls was 7.2% (92/1281), amongst ITN users 2.4% (20/842) (OR 0.31, 95% CI 0.2-0.5 P<0.001), amongst impregnated chaddar users 2.5% (18/730) (OR 0.33, 95% CI 0.2-0.6 P<0.001). Authors conclude that ITNs and impregnated chaddars were equally effective. | No significant differences for age or sex were found between new cases in the intervention and control groups. No serious side-effects reported. Interventions were generally popular; ITNs were the most popular, followed by residual  spraying and then impregnated chaddars. | 156 holes/inch^2^. Cannot be sure of compliance. About 45% of study population moved during study but this was anticipated due to violence and sample size was made adequately large with this in mind. | Yes |
| Tayeh et al, 1997. A Cutaneous Leishmaniasis Control Trial Using Pyrethroid-Impregnated Bednets in Villages near Aleppo, Syria. | Cluster randomised controlled trial. | 4578 inhabitants in 508 households. | Aleppo, Syria. | 2 intervention villages with deltamethrin impregnated bednets, 2 control villages with untreated nets. | Leishmanin skin test. | Incidence of ACL. |  | In intervention area - incidence went from 5.1% (103/2035) (pre-intervention) to 1.2% (21/1769) in the third post-intervention year. In the control area, ACL incidence doubled in the first year (from 2.4% (52/2182) to 4.6% (96/2102). In the second year went back to 2.3% (45/1985) and the third year was 6.1% (118/1929). The intervention compared to control group difference at the end of the study (after 3 years) was significant at P<0.001. | CL incidence was twice as high in households who washed their impregnated bednets once or more compared to those that did not wash them (2.0% vs 4.1% P<0.05). | Both treated and untreated bed nets had 156 holes/inch ^2^. The single person bed nets were too small to adequately cover an adult (100/800 nets distributed were affected). Heterogeneity in incidence in control villages. Third year post-intervention, one village had 1.7% (105/580) whereas the other had 8.0% (108/1349). | Yes |
| Nadim et al, 1995. Evaluation of pyrethroid impregnated bednets for control of anthroponotic cutaneous leishmaniasis in Bam (Islamic Republic of Iran). | Cluster randomised controlled trial. | 2 villages of approx 250 households each. Total of 2414 individuals. | Bam, Iran. | Deltamethrin impregnated bed nets vs no intervention. | Presence of active lesions and sores by questionnaire. | Self-reported prevalence of cases of ACL. |  | Number of cases in control area is 2.6 times higher than the intervention area (p=0.05) Intervention = 1.4% (10/728). Control = 3.1% (26/848). | Age distribution of cases - all cases in intervention area were children less than 5 years and elderly people. In the control group, all ages were seen. | Random allocation of villages to intervention/control. 10% of houses in both groups were lost to follow up. Holes/inch2 of bed nets was not mentioned. | Yes |
| Alexander et al, 1995. Laboratory and field evaluations of a repellent soap containing Diethyl Toluamide (DEET) and permethrin against phlebotomine sand flies (Diptera: Psychodidae) in Valle del Cauca, Colombia. | Randomised blind paired controlled study. | Either 4 or 2 pairs of human volunteers participated in the 9 field exposure trials. | Valle del Cauca, Colombia. | Soap containing DEET and permethrin compared to no soap or another normal soap. Two pairs applied soap immediately before exposure, the others at 4,8 or 12 hours pre-exposure. | NA | Sandfly landing rates on soaped/un-soaped areas. |  | Volunteers who applied repellent soap immediately before the assays attracted only 30.9% as many sand flies as those who applied the placebo at the same time (P < 0.05). Median numbers of sandflies collected (from skin landing) were 805 in the repellent group compared to 62.5 in the placebo group. Volunteers who applied repellent soap 4 hr before the assays attracted 44.7% as many sand flies as those using the placebo, with a median Coefficient of Protection (CP)** value of 44.3 (not significant). When soap was applied 8 hr before the assays, protected volunteers attracted  78.9% as many insects as those using the placebo, with a median CP value of 12.8. Finally, volunteers who applied the repellent soap 12 hr before exposure attracted 1.07 times as many sand flies as those using the placebo (Not significant), so that the protective effect at this time was effectively absent. | Volunteers using the placebo always attracted fewer sand flies than those who applied no treatment, with the median CP value being 67.7 and the total number captured on those using the placebo being 32.0% of that attracted to volunteers using no soap of any kind. | Authors conclude that DEET soap effective for up to 8 hr after its application in reducing the number of sandflies landing on skin. Its repellent effect is based not only on the active ingredients of DEET and permethrin, but also on the vegetable oils used in the manufacture of the soap itself. Coefficient of protection values stated cannot be calculated based on median values of sandfly capture reported in the text. | Yes |
| Alexander et al, 1995. Evaluation of deltamethrin-impregnated bednets and curtains against phlebotomine sandflies in Valle del Cauca, Colombia. | Cluster randomised controlled trial. | 2 settlements, 3 houses in each settlement assigned to each of three arms (18 houses in total). | Colombia. | Deltamethrin impregnated bednets, deltamethrin-impregnated curtains, or untreated bed nets and curtains (control). | NA | Numbers of sandflies in rooms. |  | No significant difference between the overall numbers of sandflies collected in rooms with or without impregnated curtains and bednets. Numbers of sandflies collected per man hour in control: 4.36, with impregnated curtains: 3.29 and impregnated bednets: 4.52. (no P values given). There was a significant decrease in numbers of sandflies biting under impregnated compared to untreated nets (0.26 vs 0.69 per man-hour P<0.05). |  | 413 holes/inch^2^ | No |
| Soto et al, 1995. Efficacy of permethrin-impregnated uniforms in the prevention of malaria and leishmaniasis in Colombian soldiers. | Double-blind, placebo controlled, Randomised study. | 229 subjects (Colombian soldiers). | Colombia. | Permethrin impregnated uniforms (i.e., a shirt, an undershirt, pants, socks, and a hat) or uniforms washed in water. The soldiers wore the uniforms day and night for a mean of 4.2 weeks and were observed for an additional 4 weeks. | Skin inspection and Giemsa smear as well as detection by monoclonal antibodies and biopsy smear and culture. | Cases of any Leishmaniasis. |  | In the study of leishmaniasis (soldiers were in the area of endemicity for 6.6 weeks and were observed for 12 weeks thereafter), 3% (4/143) soldiers wearing impregnated uniforms and 12% (18/143) soldiers wearing control uniforms acquired disease (P = 0.002). |  |  | Yes |

*ITN – Insecticide Treated Net

**Coefficient of Protection (CP) calculated from the formula (A-B) X 100/A. With A being the number of insects biting the untreated arm, and B being the number biting the arm treated with soap

Human vaccine studies

| Paper (author, title, year) | Study type | Number of subjects/areas | Location of study | Type of intervention | Method of diagnosis of infection | Primary outcome investigated | Secondary outcomes investigated | Main findings | Secondary findings | Comments | Intervention effective? |
| --- | --- | --- | --- | --- | --- | --- | --- | --- | --- | --- | --- |
| Khalil, 2006. Safety and immunogenicity of a candidate vaccine for visceral leishmaniasis (Alum-precipitated autoclaved Leishmania major + BCG) in children: an extended phase II study. | Phase II clinical trial. First generation vaccine.* | 544 healthy, leishmanin non-reactive children (<15yrs) were randomly allocated to receive either a single intradermal injection of Alum/ALM+BCG (273) or vaccine diluent (placebo – 271). | Sudan. | Vaccination with Alum precipitated autoclaved *L.major* (ALM) plus BCG adjuvant (intervention group) or placebo. | Not mentioned (just cases of VL were parasitologically confirmed). | LST conversion in humans. | Parasitologically confirmed human cases were reported but not a primary outcome. | Although a Phase II study which discusses safety and immunogenicity, this study as the authors report numbers of cases of VL. | During the study, 1/5% (4/271) patients in the placebo arm developed parasitologically confirmed VL. No cases of VL were reported for the vaccinated group (0/273). | Cases of VL are not an endpoint in this study. No P values are reported. | Yes |
| Velez, 2005. Failure of a killed Leishmania amazonensis vaccine against American cutaneous leishmaniasis in Colombia. | Double-blind, randomised, placebo-controlled trial. First generation vaccine. | 2597 healthy volunteers with negative leishmanin skin test (LST) randomised to receive either three doses of vaccine (n=1295) or placebo (n=1302) given at 20-day intervals. | Colombia. | Killed whole-cell *L.amazonesis* candidate vaccine plus BCG against American Cutaneous Leishmaniasis. | An active and passive case detection system was established to follow up volunteers for 1 year after vaccination. Suspected cases were confirmed by smear. | Cases of human CL. |  | 7.7% (101/1295) in the vaccine group and 6.8% (88/1302) in the placebo group developed CL. The vaccine was shown to be safe but offered no protection against CL caused by *L.panamensis* in the proposed vaccination schedule. |  |  | No |
| Armijos, 2004. Safety, immunogenicity, and efficacy of an autoclaved Leishmania amazonesis vaccine plus BCG adjuvant against New World cutaneous leishmaniasis. | Phase III clinical trial. First generation vaccine. | 1506 seronegative individuals randomised into receiving autoclaved-killed, whole cell *L.amazonesis* vaccine plus BCG adjuvant (750) or control receiving inert vaccine supernatant (756). | Ecuador. | Autoclaved-killed, whole cell *L.amazonesis* vaccine plus BCG (intervention group) or inert vaccine supernatant (control group). | A CL case was defined as the visualization of *Leishmania* parasites obtained from suspicious lesions using direct smear or aspirate culture. | Parasitologically confirmed cases of human CL. |  | The 26-month incidence of confirmed CL (n=25) was similar between the vaccine 2.0% (15/742) and placebo groups – 1.3% (10/746) P>0.005. |  |  | No |
| Armijos, 2003. Field trial of a vaccine against new world cutaneous leishmaniasis in an at-risk child population: How long does protection last? | Randomised double-blind, controlled field study. Results of a further 48 months of follow up from a previous 12-month follow up study (Armijos et al, 1998). First generation vaccine. | 1042 seronegative individuals were randomised to either the vaccine (552) or control group (487). | Ecuador. | 3 different strains of phenol-killed whole *Leishmania* promastigotes (used together as a cocktail) plus BCG adjuvant (intervention group) or BCG alone (control group). | Passive and active case detection, suspected cases were confirmed by smear. | Human clinical symptoms of CL. |  | At the end of the previous 12 months, CL incidence was significantly lower in the vaccine group (21%) compared with that in the placebo group (7.6%) P<0.003, with the protective efficacy of the vaccine calculated at 72.9% (95% CI 36.1-88.5%). During months 13-18, CL incidence remained lower in the vaccine group, compared to the control group (5.9% vs 13.8% P=0.003), with vaccine efficacy 56.5% (95% CI 18.7-76.7%); however during months 24-60, no significant difference between groups was detected. |  | Authors found that the vaccine was effective up to 18 months but after this, there was no difference. The report recommends investigation of boosters. | Yes, up to 18 months. No after 18 months. |
| Khalil, 2000. Autoclaved Leishmania major vaccine for prevention of visceral leishmaniasis: a randomised, double-blind, BCG-controlled trial in Sudan. | Double-blind randomised controlled trial. First generation vaccine. | 2306 seronegative individuals (1151 received BCG alone, and 1155 received ALM and BCG). | Sudan | Vaccination with autoclaved *L.major* (ALM) and BCG, or BCG alone. | Visual examination for clinical signs and symptoms of VL. If symptoms present, lymph node or bone marrow biopsies taken for confirmation of presence of parasites. | Human clinical visceral leishmaniasis or post kala-azar dermal leishmaniasis. |  | The cumulative frequency of VL at 2 years did not differ significantly between the group assigned ALM plus BCG and the group assigned BCG alone. 11.5% (133/1155) vs. 12.3% (141/1151) P=0.6. The vaccine efficacy was 6% (95% CI 18-25). |  | 97% of participants received both doses. | No |
| Momeni, 1999. A randomised, double-blind, controlled trial of a killed L.major vaccine plus BCG against zoonotic cutaneous leishmaniasis in Iran. | Randomised controlled trial. First generation vaccine. | 2453 healthy volunteers. | Iran. | Autoclaved *L.major* promastigotes, (ALM), mixed with BCG. | Passive and active case detection, suspected cases were confirmed by serology and parasitology. | 2 year incidence of CL in humans. |  | Cumulative incidence rates for 2 years were similar in both groups (18.0% (214/1188) for vaccine plus BCG compared to 18.5% (208/1122) for control group. |  | 92.4% follow up over 2 years. No P values reported. | No |
| Sharifi, 1998. Randomised vaccine trial of single dose of killed Leishmania major plus BCG against anthroponotic cutaneous leishmaniasis in Bam, Iran. | Randomised controlled trial. First generation vaccine. | 3637 school children aged 6-15 years, with no history of cutaneous leishmaniasis and no response to a leishmanin skin test, were randomly assigned to receive 1mg ALM mixed with BCG (n=1839) or BCG alone (n=1798). | Iran. | Vaccine consisting of a single dose of whole cell autoclave-killed *L.major* (ALM) mixed with BCG was assessed against BCG alone. | Active case finding was implemented by clinical examination of participants in schools every 2 months for a period of 2 years. Tissue smear or culture was performed on suspected cases. | 2 year incidence of CL in humans. |  | 2 year incidence of cutaneous leishmaniasis did not differ significantly between vaccine 2.8% (52/1839) and BCG group 3.3% (60/1798). |  |  | No |
| O'Daly Carbonell et al, 1995. Proteínas de amastigotes de varias especies de leishmanias  protegen a seres humanos contra la leishmaniasis en el área endémica de Guatire, Estado Miranda, Venezuela. (Spanish language paper). | Non-randomised controlled trial. First generation vaccine. | 2016 vaccinated participants both seropositive and negative, 1175 unvaccinated controls. Individuals located in 7 different communities. | Venezuela. | Vaccine comprised of 8-10 proteins from four Leishmania  Species. | Active case detection - house to house visits. Suspected cases were confirmed by ELISA and biospy for parasite visualisation. | Cases of CL. |  | The non-vaccinated control group saw 56.17 cases per thousand (66/1175) in the three years of follow up compared to 7.94 cases per thousand (16/2016) in the vaccinated group. |  | Authors state that both sero-positive and negative individuals were included in the study and that the proportion of positive (75%) to negative (25%) was similar in both groups. 668/2016 (33%) of vaccinated individuals received first dose of vaccine only, 475/2016 (24%) received 2 doses and 873/2016 (43%) received all 3 doses. Authors state a total of 2015 vaccinated individuals in the text however in tables a figure of 2016 is stated. | Yes but no P values reported. |
| Antunes et al, 1986. Controlled Field Trials of a Vaccine  Against New World Cutaneous  Leishmaniasis. | Randomised controlled trial. First generation vaccine. | 1981 study - 1312 seronegative participants (667 vaccinated, 645 placebo). 1983 study - 1274 seronegative participants (658 vaccinated, 616 placebo). | Brazil. | Vaccine consisting of killed promastigotes  from five different stocks of Leishmania. Placebo consisted of phosphate buffer. Two studies are described in the paper. One from 1981, and one from 1983. Each participant received two doses. | Bimonthly  medical (dermatological) review, suspected cases were confirmed by biopsy. | Number of cases of CL during study periods. |  | 1981 study - Group I (60 days spent in jungle - total of 611 participants). No significant difference in CL cases between vaccinated - 8.7% (28/322) and non-vaccinated - 11.1% (32/289). Group II (23 days spent in jungle - total of 701 participants). No significant difference between vaccinated - 1.2% (4/345) and non-vaccinated - 1.4% (5/356). 1983 study - no significant difference in CL cases between vaccinated - 0.6% (4/658) and unvaccinated - 1.3% (8/616). |  | Blinding and randomisation methods well described. In each study (1981 and 1983), participants were grouped according to time spent in the jungle. Group I spent 60 days whereas Group II spent 23 days. Data is reported for Groups I and II separately for the 1981 study but not for the 1983 study. No reasons for this are given by the authors. Power calculations based on estimated infection rates were conducted, however by separating participants into Groups based on time spent in jungle, adequate power was not achieved. | No |
| Mayrink, 1985. An experimental vaccine against American dermal leishmaniasis: experience in the State of Espirito Santo, Brazil. | Randomised control trial. First generation vaccine. | 782 seronegative individuals: 216 vaccinated, 266 control. | Brazil. | Vaccine prepared from killed and sonicated promastigotes of five Brazilian strains of Leishmania was used during an epidemic of American dermal leishmaniasis. | Clinical appearance of lesions with parasite confirmation by smear. | Clinical symptoms of CL in humans verified by parasitology. |  | 1.5% of those vaccinated and 6.4% of the unvaccinated group developed dermal leishmaniasis by the end of the first year. At the end of the second year, 1.7% (3/179) of those vaccinated and 8.9% (18/203) of the unvaccinated group had become infected. The difference in infection rates of the two groups is statistically significant at the end of both the first and second year of observation. |  | Total loss to follow up from both groups was 20.7%. | Yes |
| Mayrink, 1979. A field trial of a vaccine against American dermal leishmaniasis. | Randomised control trial. First generation vaccine. | 614 individuals vaccinated, 974 unvaccinated controls. | Brazil. | Vaccine containing killed promastigotes of five stocks of Leishmania. | Passive case detection. | Incidence of CL in vaccinated and unvaccinated groups. |  | No cases of cutaneous or mucocutaneous leishmaniasis occurred in the trial area during the three years of observations. |  |  | Inconclusive- no cases in either group. |

*First generation vaccine defined as fractions of the parasite or whole killed Leishmania with or without adjuvants.
