# Supplementary material for: A Review of Preventative Methods against Human Leishmaniasis Infection
Source: PLoS Negl Trop Dis. 2013 Jun 20;7(6):e2278. doi: 10.1371/journal.pntd.0002278 (PMC3688540; doi:10.1371/journal.pntd.0002278)
Supplement: Dataset S4 — Full dataset of Multiple interventions studies. (DOCX) [file pntd.0002278.s004.docx]

Multiple Interventions

| Paper (author, title, year) | Study type | Number of subjects/areas | Location of study | Type of intervention | Method of diagnosis of infection | Primary outcome investigated | Secondary outcomes investigated | Main findings | Secondary findings | Comments | Intervention effective? |
| --- | --- | --- | --- | --- | --- | --- | --- | --- | --- | --- | --- |
| Souza et al, 2008 Communitary assay for assessment of effectiveness of strategies for prevention and control of human visceral leishmaniasis in the Municipality of Feira de Santana, State of Bahia, Brazil. (Portuguese language paper). | Controlled trial. | 3 villages - containing 688, 782 and 892 infants (9 mo - 12 yrs) respectively. | Feira de Santana, State of Bahia, Brazil. | Control (as usual - not specified) compared to spraying with pyrthroid insecticide and spraying with dog culling (max 15 days post diagnosis). | ELISA | Incidence of infection of VL in children. |  | Relative risk for infection in the areas with the combination of spraying and dog culling compared to control = 0.74 (95% CI 0.34-1.62). Although the data suggest a reduction of the incidence of infection in the intervention areas, this difference was not statistically significant. |  | No P values given. | No |
| Moosa-Kazemi et al, 2007. Deltamethrin-impregnated bed nets and curtains in an anthroponotic cutaneous leishmaniasis control program in northeastern Iran. | Cluster randomised controlled trial. | Total of 1,848 residents. 160 x 3 households. | Iran. | Deltamethrin-impregnated bed nets and curtains vs untreated bed nets and curtains (control, no intervention). | Case finding by house to house visits and examination of all inhabitants and smears taken. | Incidence of ACL in 2004. | Sandfly density. | Impregnated bed nets and curtains vs non-impregnated bednets and curtains incidence per 1000 = 6.9 vs 30.6 (P=0.028). Impregnated bednets and curtains vs control incidence; 6.9 vs 28.6 (P=0.025). Actual numbers of lesions in each group post-intervention: Impregnated bed nets and curtains - 2/580. Non-impregnated bed nets and curtains - 11/569, and control group 18/699. | No significant difference in monthly sandfly densities between villages. | Net with 156 holes/inch^2^ was used. From the numbers of lesions quoted in the tables, it was unclear how the authors arrived at the incidence rates quoted per 1000 in the report. | Yes |
| Nery Costa et al, 2007. Controle da leishmaniose visceral em meio urbano: estudo de intervenção randomizado fatorial. (Portuguese language paper). | Randomized factorial intervention trial with 3 arms. | 367 individuals were tested for serological Leishmaniasis. The area of Teresina, BR, was divided in 34 blocks randomly allocated to 4 types of intervention. | Brazil. | 1) spraying houses and animal pens with insecticide; 2) spraying houses and  eliminating infected dogs; 3) combination of spraying houses and animal pens, and eliminating infected dogs, and 4) only spraying houses (control). | Human seroprevalence ( by ELISA). Dog seroprevalence (by Indirect immunofluorescence - IFAT) used to detect seropostive dogs to be eliminated. | Human and dog infection - measured by seroprevalence. |  | Spraying houses and eliminating infected dogs (arm 2) was associated with a decreased incidence of VL compared to ‘no intervention’ control (which actually consisted of house spraying only) OR 0.2 (95% CI 0.04-0.89). Spraying houses, animal pens and eliminating dogs (arm 3) was associated with a decrease in VL compared with the control group. OR 0.69 (95% CI 0.27-1.76). No P values reported. Baseline prevalence in 367 individuals across all areas was 42%. Of the remaining 213 seronegatives, only 56% (120) were analysed after 6 months to test seroconversion. Of these 16.1% became infected during 6 months after being in the spraying with dog elimination group, and 37.9% of the house and animal pens as well as dog culling intervention became infected. |  | No P values reported. The 44% of drop outs were not described by intervention group and therefore it is impossible to differentiate the denominator of how many seropositives there were in each group. Only percentages are reported. The OR values reported for intervention groups 2 and 3 infer that adding the spraying of animal pens actually decreases the protective effect, indicating that this result may be due to chance. | The spraying of houses plus animal culling is effective (OR 0.2) whereas including spraying of animal pens as well, decreased the protective effect (OR 0.69). |
| Rojas et al, 2006. A multifaceted intervention to prevent American cutaneous leishmaniasis in Colombia: results of a group-randomized trial. | Cluster randomised controlled trial. | 20 villages matched according to prevalence of Leishmania infection, number of inhabitants and level of community participation, and then randomly assigned to intervention or control. | Tumaco, Colombia. | Intervention included deltamethrin-impregnated bednets, use of repellent in surrounding forest (diethyltoluamide and permethrin), modification of sand fly resting sites (white washing trees), and health education vs no intervention (control). | Active case finding -examination for scars and active lesions. LST was used to detect previous infection. | Human incidence of ACL. | LST conversion was also investigated but not all participants were tested. | 0.56% (10/1719) in the intervention group compared to 1.25% (23/1840) in the control group OR = 0.42, 95% CI 0.14-1.26 (not significant, no P value given). | LST conversion was 82/1066 (only 1066/1719 were tested) in the intervention group, and 80/1034 (only 1034/1840 were tested) in the control group. Adverse events associated with the use of the bednets and the repellent were reported in 2% of the participants and were always mild. | Randomisation explained. The small number of cases renders the effect estimate imprecise and precludes a claim of a protective effect for the intervention. | No |
