# Supplementary material for: A Review of Preventative Methods against Human Leishmaniasis Infection
Source: PLoS Negl Trop Dis. 2013 Jun 20;7(6):e2278. doi: 10.1371/journal.pntd.0002278 (PMC3688540; doi:10.1371/journal.pntd.0002278)
Supplement: Text S1 — Search strategy for database searching of Medline, EMBASE, CENTRAL, Web of Science, LILACS and WHOLIS. (DOCX) [file pntd.0002278.s005.docx]

**OVID MEDLINE search terms**

General search to identify randomised controlled trials, clinical trials and crossover studies relating to Leishmaniasis:

1. exp Leishmaniasis/ or leish*.mp.

2. kala azar.mp.

3. 1 or 2

4. trial*.mp,ti.

5. clinical trial.ab,ti.

6. RANDOMIZED CONTROLLED TRIAL.pt.

7. controlled clinical trial.pt.

8. random allocation.sh.

9. randomized controlled trial.sh.

10. double blind method.sh.

11. single blind method.sh.

12. CLINICAL TRIAL.pt.

13. exp Clinical Trial/

14. (clin* adj25 trial*).ti,ab.

15. PLACEBOS.sh.

16. placebo*.ti,ab.

17. random*.ti,ab.

18. ((singl* or doubl* or trebl* or tripl*) adj25 (blind* or mask*)).ti,ab.

19. exp Cross-Over Studies/

20. crossover.ti,ab.

21. CROSS-OVER-STUDIES.sh.

22. intervention study.mp. or exp Intervention Studies/

23. 4 or 5 or 6 or 7 or 8 or 9 or 10 or 11 or 12 or 13 or 14 or 15 or 16 or 17 or 18 or 19 or 20 or 21 or 22

24. 3 and 23

For dog culling studies the following search terms were added to the general search terms:

25. cull*.ab,ti.

26. (dog* adj25 cull*).ti,ab.

27. animal euth*.mp.

28. canine cull*.mp. or exp Euthanasia, Animal/

29. population control.mp. or exp Population Control/

30. 25 or 26 or 27 or 28 or 29

31. 24 and 30

For insecticide-impregnated dog collars the following search terms were added to the general search terms:

32. impregnated dog collar*.mp.

33. (dog* adj25 collar*).ab,ti.

34. 32 or 33

35. 24 and 34

For household residual spraying interventions the following search terms were added to the general search terms:

36. residual spray*.mp.

37. house* spray*.mp.

38. 36 or 37

39. 24 and 37

For environmental studies the following search terms were added to the general search terms:

40. environmental manipulation.mp.

41. environmental management*.mp.

42. building materials.mp. or exp Construction Materials/

43. housing.mp.

44. insect control.mp.

45. 40 or 41 or 42 or 43 or 44

46. 24 and 45

For vaccine trials the following search terms were added to the general search terms:

47. vaccin*.mp. or exp Vaccines, Conjugate/

48. exp Vaccination/ or vaccination.mp.

49. 47 or 48

50. 24 and 49

**OVID EMBASE search terms**

General search to identify Leishmaniasis clinical trials:

1. trial*.ab,ti.

2. leishmania*.mp. or exp Leishmania/

3. kala azar.mp.

4. 2 or 3

5. 1 and 4

For dog culling the following search terms were added to the general search terms:

6. exp animal euthanasia/

7. dog culling.mp.

8. cull*.ab,ti.

9. (dog* adj25 cull*).ti,ab.

10. (canine* adj25 control*).ti,ab.

11. (canine* adj25 removal*).ti,ab.

12. animal euth*.mp.

13. canine cull*.mp. or exp Euthanasia, Animal/

14. population control.mp. or exp Population Control/

15. 6 or 7 or 9 or 10 or 11 or 12 or 13 or 14

16. 5 and 15

For environmental management interventions the following search terms were added to the general search terms:

17. environmental manipulation.mp.

18. building materials.mp. or exp Construction Materials/

19. exp environmental management/ or environmental manage*.mp.

20. vector control.mp. or exp vector control/

21. 17 or 18 or 19 or 20

22. 5 and 21

For household residual spraying interventions the following search terms were added to the general search terms:

23. residual spray*.mp.

24. house* spray*.mp.

25. 23 or 24

26. 5 and 25

For general insecticide interventions the following search terms were added to the general search terms:

27. exp insecticide/ or insecticide.mp.

28. exp permethrin/ or exp pyrethroid/ or permethrin-impregnated.mp.

29. 27 or 28

30. 5 and 29

For dog collars the following search terms were added to the general search terms:

31. exp insecticide/ or insecticid*.mp.

32. (topical* adj25 application*).ab,ti.

33. insecticide-impregnated dog collars.mp.

34. 31 or 32 or 33

35. dog*.mp.

36. canine.mp.

37. 35 or 36

38. 34 and 37

39. 5 and 38

For vaccine trials, the following search terms were added to the general search terms:

40. exp vaccination/ or vaccin*.mp. or exp vaccine/

41. 5 and 40

**Web of Science Search terms**

1. Topic=(leishmania* OR kala azar) AND Topic=(trial* OR random* OR controlled)
2. Topic=(environment* manage* OR building material* OR hous*)
3. Topic=(cull* OR animal euthanasia)
4. Topic=(impregnated dog collar*)
5. Topic=(residual spray* OR house spray*)
6. Topic=(vaccine*)
7. #6 OR #5 OR #4 OR #3 OR #2
8. #7 AND #1

**Cochrane Central Register of Controlled Trials (CENTRAL) Search terms**

CENTRAL was searched using the terms "leish* AND (prevent* OR control OR vaccin* OR manage*) in Title, Abstract or Keywords.

**LILACS Search terms**

Search in title: “(leishmania* OR kalaazar) AND (prevent* OR vaccine* OR manage*)”.

**WHOLIS (from 1992) Search terms**

Separate searches were carried out using the following;

words or phrase “Leishmania”, “Leishmaniasis”, “kalaazar”.
